# Versatile Preparation of Branched Polylactides by Low-Temperature, Organocatalytic Ring-Opening Polymerization in *N*-Methylpyrrolidone and Their Surface Degradation Behavior

**DOI:** 10.1021/acs.macromol.1c01503

**Published:** 2021-10-07

**Authors:** Giulia Scoponi, Nora Francini, Veronica Paradiso, Roberto Donno, Arianna Gennari, Richard d’Arcy, Carmine Capacchione, Athanassia Athanassiou, Nicola Tirelli

**Affiliations:** † Smart Materials, Istituto Italiano di Tecnologia, Via Morego 30, 16163 Genova, Italy; ‡ DIBRIS, University of Genova, Via Opera Pia 13, 16145 Genova, Italy; § Laboratory of Polymers Biomaterials, Istituto Italiano di Tecnologia, Via Morego 30, 16163 Genoa, Italy; ∥ Department of Chemistry and Biology “Adolfo Zambelli”, University of Salerno, Via Giovanni Paolo II 132, 84084 Fisciano, Italy; ⊥ School of Health Sciences, University of Manchester, Oxford Road, M13 9PL Manchester, U.K.

## Abstract

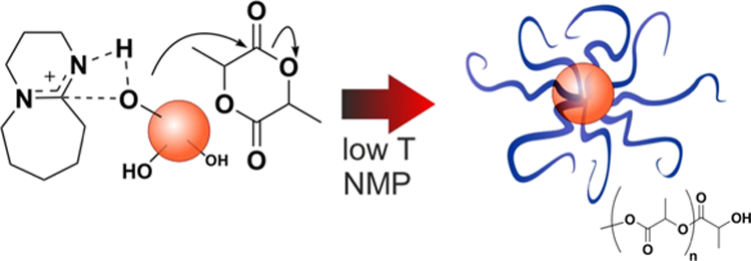

We describe how the organocatalytic, 1,8-diazabicyclo[5.4.0]undec-7-ene (DBU)-based lactide ring-opening polymerization can be effectively performed in a very polar solvent, *N*-methylpyrrolidone (NMP). Due to a low ceiling temperature, this “living” mechanism has been unreported to date, but we here demonstrate that through a combination of low temperature and repeated monomer additions (starve-fed process), this mechanism enables the generation of a plethora of multifunctional homo- and (stereo)block-poly(lactide)s (PLAs) with exquisite control of the molecular weight dispersity (typically Đ < 1.1) and topology (from linear through 4-, 6-, or 8-armed stars and up to ∼140 armed combs). They are scarcely obtainable or inaccessible through more classical synthetic methods due to the poor solubility of multifunctional initiators (polyols) in most organic solvents and monomer melts. In these precisely designed structures, branching significantly altered the nature of the materials’ hydrolytic degradation, allowing them to acquire a pronounced surface character (as opposed to the bulk degradation of linear polymers). Finally, we have assessed the amenability of this method to in situ block copolymerization by using the tacticity of PLLA blocks in PLLA-*b*-PDLLA *versus* PDLLA-*b*-PLLA (L-LA polymerized before or after DL-LA) as a sensitive method to detect (stereochemical) defects.

## Introduction

Traditionally, and to date probably also more commonly than not, the ring-opening polymerization (ROP) of lactides, glycolides, and other cyclic esters makes use of metallorganic catalysts, such as tin octoate. Typical drawbacks of such processes are (1) transesterification reactions occurring during and possibly after the polymerization, which increase the molecular weight (MW) dispersity and decrease the control over the identity of terminal groups; (2) the non-negligible presence of tin in the final polymers; (3) the typically rather high temperatures required (possible racemization); and (4) the necessity to conduct the reactions in bulk or in hydrophobic solvents: the former is a greener approach but casts strong limitations in terms of solubility and parasite reactions, while the latter is less environmentally friendly and still limited in terms of solubilization of functional monomers and initiators.

Star polyesters offer probably the best example of a controlled branched architecture;^1^ their finely tuned structures have been used to highlight that branching appears to promote a surface-dominated degradation mechanism (*vs* the bulk degradation of linear polyesters^2^) and offers a wealth of possibilities to modulate mechanical, rheological, and thermal properties *via* their branching degree. Their preparation through traditional (*e.g.*, tin octoate-based) ROP using polyols as initiators is sometimes possible but inconvenient. The products are typically marred by a broad MW distribution,^3,4^ which is in part due to different OH groups in a given polyol initiating at different times to the point of some possibly not taking part in the reaction^5^ and leading to “comet”-shaped instead of star-shaped macromolecules.^6^ Further, polyols with a large number of OH groups are poorly soluble in the reaction environment of classical ROP; for example, hexa-functional dipentaerythritol (diPET) and octa-functional tripentaerythritol are not soluble in the lactide monomer, and tripentaerythritol is not soluble in toluene.^5^ In order to prepare well-defined star polyesters, it is therefore advantageous to employ alternative polymerization methods.

In the last 20 years, due to the possibility of using lower temperatures and (polar) solvents, organocatalytic ROPs have become increasingly popular alternatives, in particular, those employing bases structurally derived from amidine or guanidine, for example, 1,8-diazabicyclo[5.4.0]undec-7-ene (DBU, from amidine) and 1,5,7-triazabicyclo[4.4.0]dec-5-ene or 7-methyl-1,5,7-triazabicyclo[4.4.0]dec-5-ene (respectively, TBD or MTBD, from guanidine).^7,8^ In a first approximation, one could assume these bases to promote a form of living anionic mechanism (or pseudoanionic, when, *e.g.*, TBD also acts as a hydrogen bonding donor^9^); there, propagating species are alcoholates and protonated bases, acting as counterions, modulate the polymerization kinetics. DBU offers a “Goldilocks” compromise between the excessively rapid TBD, which is also marred by transesterification, and the too slow MTBD.^7,10^

**Scheme 1:**
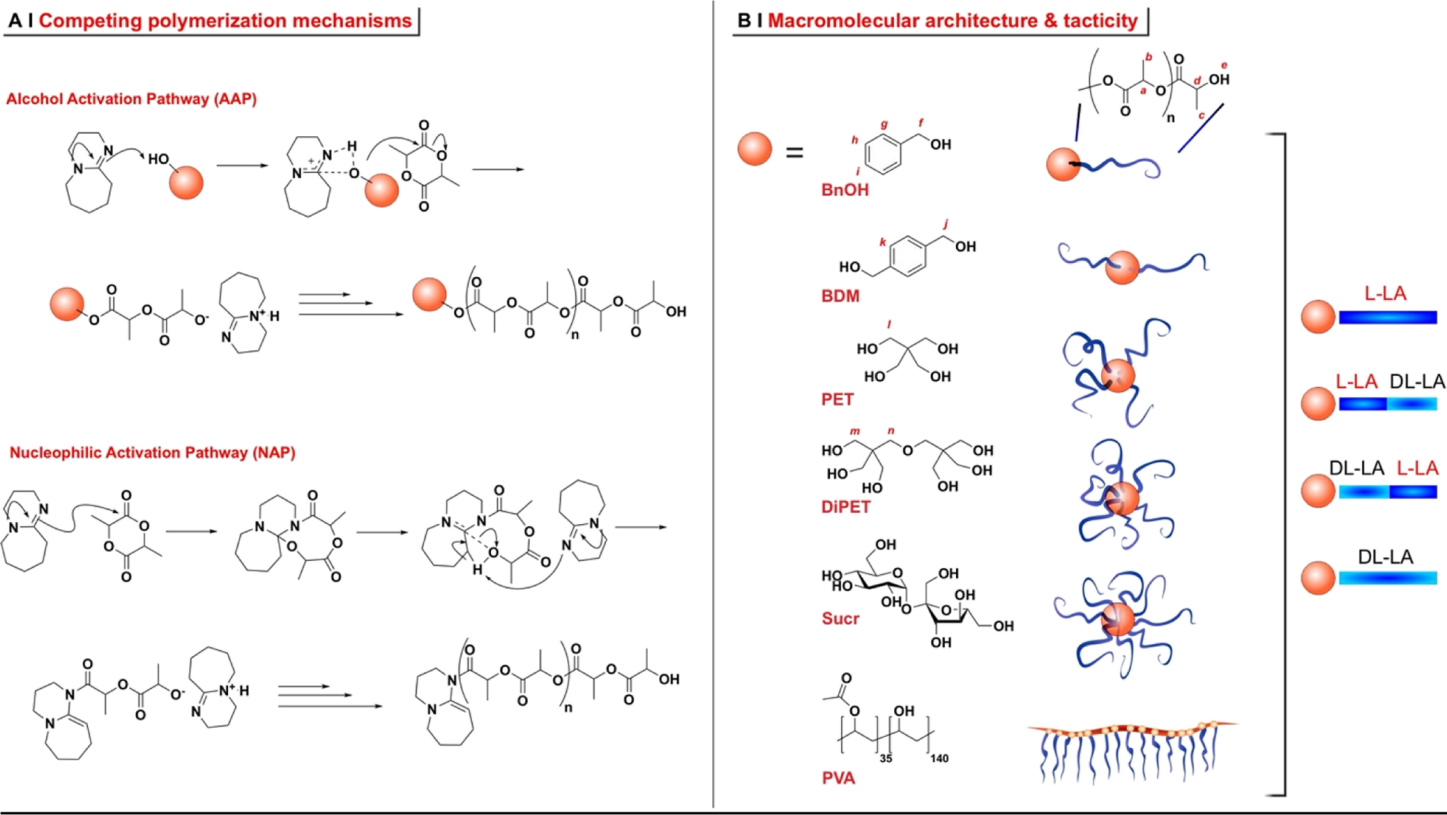
(A) Two Potentially Competing Mechanisms Are Operational in the DBU-Activated Lactide Polymerization;^11^ (B) Initiators with Variable Functionality Were Employed in This Study, Namely, Benzyl Alcohol (BnOH), 1,4-Benzendimethanol (BDM), Pentaerythritol (PET), Dipentaerythritol (diPET), Sucrose (Sucr), and Poly(vinyl alcohol) (PVA) Atom numbering in red refers to the ^1^H NMR assignments (see the Materials and Methods section); please note that for sucrose, no resonance could be clearly recognized as part of the initiator structure. Depending on the functionality, linear and star/comb poly(lactide) architectures can be produced; both L-LA and DL-LA were employed as monomers, either alone to yield “homopolymeric” arms or in a sequence to yield “blocky” arms with the crystallizable monomer (L-LA) sequences close or distant from the branching point.

A partial drawback of these base-activated organocatalytic ROPs is the possibility of initiation through multiple mechanisms. For example, it has been shown that DBU (Scheme 1A) may initiate both by deprotonating the alcohol group(s) of an initiator or by directly activating the monomer; according to the definition of Sherck *et al.*;^11^ the former is the alcohol activation pathway (AAP), while the second is known as the nucleophilic activation pathway (NAP). In particular, the NAP is a problem in the synthesis of star structures: a multifunctional initiator (a polyol) may be used, yet a significant amount of the linear polymer will be obtained instead. If the NAP can be minimized, for example, using alcohols in stoichiometric equivalence or even excess with respect to DBU,^11^ then organocatalytic ROP becomes an attractive method to yield star polymers. Indeed, the use of polar solvents can better solubilize polyol initiators, and low temperatures not only entail lower energetic costs and a lower likelihood of racemization but also, due to the equilibrium nature and the negative enthalpy of lactide ROP, minimize the concentration of residual monomer ,^12^ thereby maximizing the yield of the polymerization process. Conversely, high temperatures have the opposite effect, to the limit of no polymerization at all at the so-called ceiling temperature (*T*_c_). As an example, a monomer such as γ-butyrolactone, long considered to be “unpolymerizable” due to low ring strain,^13^ has now been successfully polymerized, with a majority of methods using sub-zero (below *T*_c_) temperatures.^14,15^

The maximization of monomer conversion can be an advantageous point to produce branched polyesters with long arms. Indeed, apart from tin octoate and related techniques, which are typically marred by poor MW dispersity, methods to produce long-armed structures are still lacking; for example, high-MW star polyesters are most often obtained using highly multifunctional polyols such as Boltorn^16^ or hyperbranched poly(ethylene glycol),^17^ with arms typically bearing no more than 20 monomeric units.

In this study, we have focused on the possibility of using *N*-methylpyrrolidone (NMP) as a polymerization solvent. First, NMP is “greener” than the more commonly used chloroform, dichloromethane, or toluene: for example, NMP is well-tolerated *in vivo* and indeed commonly exploited as a transdermal penetration enhancer,^18^ possibly even effective in reducing vascular inflammation^19^ and degradable by selected bacterial strains;^20^ NMP is also EPA-approved for food and nonfood use,^21^ although potential risks for water environments have recently been raised.^22^ Second, NMP can dissolve a wide variety of functional molecules, including polyols from low-MW compounds to macromolecules such as polysaccharides or poly(vinyl alcohol) (PVA). However, it is not known if NMP may have negative effects on lactide polymerization; for example, its higher polarity may affect the balance between the AAP and NAP or—even more profoundly—the very equilibrium nature of the ROP. Indeed, the better monomer solvation provided by a more polar solvent may reduce its ring strain and therefore also the enthalpic driving force for polymerization, as seen for the DBU-assisted six-membered carbonate polymerization in acetonitrile *versus* toluene.^23^

We have then used a series of polyols with increasing functionality (Scheme 1B) to trigger the DBU-catalyzed ROP of lactide in NMP. The presence and functionality of the branching points may have a profound effect on the ordered assembly, for example, crystallization of the poly(lactide) (PLA) arms. Therefore, we have produced block copolymers of d,l-lactide (DL-LA) and l-lactide (L-LA), where poly(l-lactide) PLLA crystallizable blocks are proximal or distal to the branching point, that is, respectively, more or less sterically hindered by it; poly(d,l-lactide) PDLLA and PLLA homopolymeric arms were also produced as controls.

## Materials and Methods

### Materials

DL-LA, L-LA, DBU, acetic acid, benzyl alcohol (BnOH), pentaerythritol (PET), diPET, sucrose (Sucr), and PVA (80% hydrolyzed,  = 13 kDa, Đ =1.3) were purchased from Sigma-Aldrich (Merck Life Science, IT). NMP and 1,4-benzenedimethanol (BDM) were purchased from Fluorochem (UK).

### Purification Procedures

All reagents (monomers, initiators, catalysts, and the solvent) were carefully purified to remove traces of water; this is important to avoid initiation or ester hydrolysis by OH anions produced by water in the presence of the strongly basic DBU. DL-LA and L-LA were recrystallized twice in dry toluene, dried under vacuum, and stored under an inert atmosphere until needed. BnOH was distilled at 120 °C, 90 mbar. BDM was recrystallized twice from dry CHCl_3_ and then dried in a vacuum oven (around 0.1 mbar) at 40 °C for 2 days. PET was sublimated at 240 °C and 60 mbar and stored under reduced pressure. In order to remove water, DiPET and PVA dispersed in toluene were introduced in a round-bottomed flask and refluxed over a Soxhlet apparatus filled with 3 Å molecular sieves for 3 h (water removed as the minimum azeotrope is trapped by the molecular sieves); the solvent was then removed under vacuum, and dry DiPET and PVA remained at the bottom of the flask; and they were used within 12 h by adding the appropriate polymerization solvent to the same flask and thereby producing stock solutions of the two initiators. Sucrose was freeze-dried and subsequently kept in a vacuum oven (0.1 mbar, 35 °C) for 48 h prior to use. DBU was vacuum-distilled at 85 °C, 0.6 mbar and stored under reduced pressure for up to 2 weeks; NMP was stirred overnight with calcium hydride and subsequently vacuum-distilled (75 °C, 5 mbar).

### Physicochemical Characterization

#### Gel Permeation Chromatography/Size-Exclusion Chromatography

Gel permeation chromatography (GPC)/size-exclusion chromatography (SEC) was performed on an integrated OMNISEC system (Malvern PANalytical Ltd., UK) equipped with a D6000M and a D4000 column (10 and 6 μm particle sizes, respectively, 300 × 8 mm) using a triple-detection method (refractive index, viscometer, and dual-angle light scattering detector at 7 and 90°). Tetrahydrofuran (THF) stabilized with 250 ppm butylated hydroxytoluene was used as the eluent at a temperature of 35 °C and a flow rate of 1.0 mL/min. The system was calibrated with the polystyrene (PolyCAL Standards, Malvern PANalytical Ltd., UK) 105 kDa narrow standard and verified with a 250 kDa broad standard of known dispersity, intrinsic viscosity, and d*n*/d*c*. Data analysis was performed using OMNISEC software V11.10. Prior to each analysis, samples were dissolved in THF at a known concentration and room temperature (RT), and the resulting solutions were filtered through a 0.22 μm poly(tetrafluoroethylene) (PTFE) filter. Triple detection was used to obtain absolute MW distributions, intrinsic viscosity, hydrodynamic radius (*R*_H_), and Mark–Houwink parameters (log *K* and *a*) of the PLAs. The radius of gyration *R*_g_ was not calculated as the synthesized PLAs have too low d*n*/d*c* (d*n*/d*c* data are shown in the Supporting Information) and hydrodynamic size to produce anisotropic scattering, which is required for *R*_g_ calculations.

#### Static Light Scattering

A 5.0 mg/mL stock solution of each polymer [*i.e.*, PVA-*comb*-PLLA, PVA-*comb*-PDLLA, PVA-*comb*-(PLLA-*b*-PDLLA), and PVA-*comb*-(PDLLA-*b*-PLLA)] in THF was filtered through a 0.2 μm PTFE syringe filter and then diluted with the same solvent in order to produce five concentrations ranging between 1.5 and 5.0 mg/mL. 1.5 mL of each solution was injected into a DAWN HELEOS II multi-angle light scattering detector, operating at 660 nm and 25 °C (Wyatt Technology, US), using a syringe pump (Kent Scientific Corporation, US). Software Astra version 7.1.4.8 (Wyatt Technology, US) was used to collect and analyze the static light scattering (SLS) data (Zimm plots; see Supporting Information, Figure S5) and obtain the weight-average MW, radius of gyration (*R*_g_), and second virial coefficient (A2) using the nominal concentration of the solutions and the d*n*/d*c* data previously obtained for the polymers with the same composition analyzed by GPC/SEC. All samples were analyzed using the Debye formalism with the fit degree equal to 1 for both the angle and concentration.

#### Nuclear Magnetic Resonance

Evaluation of the monomer conversion and polymer chemical structure was carried out by ^1^H NMR experiments, performed on a Bruker AVANCE III 400 MHz spectrometer (Bruker Ltd., US) equipped with a broadband inverse probe and Z-gradients. The polymers were dissolved at a concentration of 10 mg/mL in deuterated CHCl_3_. ^1^H NMR spectra are referenced using the residual solvent peak at δ 7.26 ppm. Measurements were performed at 27 °C. Results were analyzed using MestreNova (Mestrelab Research S.L., ES) software.

For the evaluation of polymer tacticity, ^1^H NMR spectra were acquired on ∼10 mg/mL polymer solutions in deuterated CHCl_3_ on a Bruker Ascend 600 spectrometer. The methyl protons (δ ∼ 1.5 ppm) were decoupled from the methine protons (homonuclear decoupling) during the acquisition time.^24,25^

The sequences of stereocenters in the macromolecular chains are interpreted on the basis of the possible combinations of *m* (“meso” or isotactic, *i.e.*, pairwise relationship −RR– and −SS−) and *r* (“racemic” or syndiotactic, *i.e.*, −RS– and −SR−) diads.^26^ The probability P*r* to obtain an *r* diad (ranging between 0 for fully isotactic and 0.5 for atactic PLA structures) is calculated as
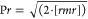
where [*rmr*] is the concentration of an *rmr* tetrad, which is calculated from the relative intensities of methine signals; for an explanation of the formula, please refer to its Bernoullian statistics derivation by Vert.^27^ After homonuclear decoupling, tetrads *mrm*, *mmm*, and *mmr* and hexads *mmmrr*, *mmmrm*, and *mrmrm* can be recognized in the ^1^H NMR spectra, respectively, with resonances at δ = 5.18, 5.19, 5.20, 5.22, 5.23, and 5.24 ppm; since only the last hexad contains the *rmr* tetrad, the corresponding area can be used to calculate [*rmr*] as follows
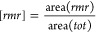


The percentage of racemization (%rac) was calculated by the deconvolution of the methine multiplet of the ^1^H NMR homonuclear decoupled spectrum using the Mestrelab software fitting tool.

#### Differential Scanning Calorimetry

Differential scanning calorimetry (DSC) thermograms were acquired with a Q2500 (TA Instruments, US) DSC system equipped with a RCS-90 refrigerated cooling system. Powder polymer samples (∼5 mg) were loaded into hermetic aluminum pans, and a conventional heating–cooling–heating scan was performed between −20 and 180 °C with a temperature ramp of 10 °C/min under a nitrogen atmosphere. Equilibration isothermal periods of 2 min were applied prior to each scan. Melting (*T*_m_) and crystallization (*T*_c_) temperatures were measured as the maxima of the corresponding phase transition peaks; in some instances, it was useful differentiate the location of the onset and that of the maximum of the melting process, and on these occasions, *T*_m|onset_ and *T*_m|max_ were correspondingly used. The glass transition temperatures (*T*_g_) were calculated as the inflection points of the transition in the second heating run. Melting enthalpy (Δ*H*_m_) and, if present, cold crystallization enthalpy (Δ*H*_cc_) were determined by the integration of the corresponding transition peaks in the first heating DSC thermograms. Crystallinity (*X*_c_) was subsequently calculated as follows
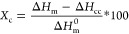
where Δ*H*_m_^0^ is the enthalpy of fusion of 100% crystalline PLLA (93 J/g).^28^

#### X-ray Diffraction Analysis

X-ray diffraction (XRD) measurements of multiarmed PLA powder samples were performed on a PANalytical Empyrean X-ray diffractometer using a Cu Kα anode (λ = 1.5406 Å) operating at 45 kV and 40 mA. The diffraction patterns were collected in the range 2–70° 2θ with a 0.04° step size.

#### Shear Rheometry

Measurements were performed on 6-armed PLA samples using a HAAKE MARS 40 rheometer (Thermo Fisher Scientific, US) equipped with a flat probe P35/Ti (parallel plate geometry). The storage modulus (*G*′) and loss modulus (*G*″) were measured as a function of temperature, which was linearly decreased (Δ*T* = 5 °C/s) from 200 to 25 °C, by applying a shear stress and an oscillation frequency (*f*) of 25 Pa and 1 Hz, respectively. Once the complex modulus is defined as , the complex viscosity is obtained as η* = G*/(2π*f*); the phase angle δ is defined as δ = arc tan(*G*″/*G*′).

#### Atomic Force Microscopy

##### Sample Preparation

Homogeneous polymer films were produced by drop-casting 20% w/v CHCl_3_ solutions of 6-armed PLAs on Ø = 13 mm circular glass coverslips, employed as a rigid support. The solvent was slowly removed by leaving samples at ambient pressure and RT for 2 days, followed by 24 h under reduced pressure and RT in a vacuum oven. Subsequently, films were annealed at 80 °C for 10 h in order to reach the maximum crystallinity depending on the polymer stereoconfiguration.

##### Measurements

Nanoindentation studies were performed in air using a molecular force probe 3D atomic force microscope (Model MFP-3D, Asylum Research—Oxford Instruments, UK). A TESPA-V2 sharp tip (Bruker AFM Probes, US) was used for all measurements. The actual spring constant (*k*) was determined by the thermal noise method, *k* = 33 N/m. On each sample analyzed, several force maps were acquired, performing 36 indentation curves (maximum force applied = 1 μN) within a 20 μm × 20 μm area (spatial resolution ≈ 11 μm^2^) at 0.25 Hz (0.75 μm/s). Corresponding force maps were acquired. The relative elastic modulus was calculated by fitting the force indentation data with the Hertz sphere model (Hertz sphere-on-flat model).

#### Degradability

The hydrolytic degradation of PLAs was monitored by following the MW distribution (degradation) and weight loss (erosion) over a 50 day period of incubation in a phosphate-buffered saline (PBS) medium. *Experiments on polymer particulates*: ∼15 mg of 1-armed (PLLA and PDLLA) and 4-armed (PLLA, PLLA-*b*-PDLLA, PDLLA-*b*-PLLA, and PDLLA) polymer powders (directly from precipitation after drying in a vacuum oven) was introduced in Eppendorf tubes, and 2 mL of PBS was added and left at 37 °C under gentle agitation in an orbital shaker for 0, 4, 14, 24, 34, and 50 days. Once their incubation was concluded, samples were centrifuged (14,000*g*, 10 min) and washed with Milli-Q water. The resulting polymer pellets were freeze-dried, weighed, and finally dissolved in 2 mL of THF for GPC/SEC analysis. All experiments were performed in triplicate. *Experiments on polymer films*. 50 mg of 40% w/v polymer solutions in CHCl_3_ of L_(PLLA) and S_(PDLLA)_4_ was casted on Ø = 14 mm circular glass coverslips. The solvent was gradually removed through a stepwise reduction in pressure (from room pressure to 1 mbar in 6 h, followed by 48 h at 1 mbar) in a vacuum oven at 40 °C. The supported films were placed in 24-well polystyrene plates and incubated in 2 mL of Milli-Q water at 37 °C under gentle agitation for different periods of time: 0, 6, 12, 21, 30, 40, and 50 days. The samples were then washed with Milli-Q water, dried under reduced pressure for 48 h at 35 °C, weighed, and analyzed as per the analysis of samples from particulates.

#### Scanning Electron Microscopy

The polymer particulates used in degradation experiments were characterized by scanning electron microscopy (SEM), using a JEOL JSM-6490LA microscope (JEOL Ltd., JP) working in a high-vacuum mode with an acceleration voltage of 10 kV. The samples were previously deposited on metal stubs and then coated with a 10 nm gold layer by using a high-resolution sputter coater Cressington 208HR (Cressington Scientific Instruments Ltd., UK). The particulate size was assessed by processing the SEM images using ImageJ (Wayne Rasband National Institutes of Health, USA).

### Synthetic Procedures

All experiments were performed in a 12-position Carousel parallel reactor (Radleys, UK), heated under vacuum and subsequently purged with nitrogen for 5 min prior use. In a typical polymerization, the final number-average degree of polymerization per arm () was set to be 100, and the monomer concentration was maintained at 1 M. Four sets of experiments were performed in the presence of different multifunctional initiators: (1) L-LA alone, (2) DL-LA alone, (3) L-LA first (50% of the monomer feed) and then DL-LA, and (4) the reverse of (3). BnOH-initiated PLA was produced for every set of experiments, as a linear polymer reference.

First, stock solutions of DL-LA and L-LA (1.4 M) and initiators (140, 70, 35, 23.3, 17.5, and 9.5 mM for BnOH, BDM, PET, and diPET, Sucr, and PVA, respectively) in dry NMP were prepared under an inert atmosphere. Subsequently, 2 mL of the monomer and 400 μL of each initiator stock solution were transferred into the corresponding Carousel vessels and equilibrated at 5 °C using an ice bath. The total amount of the desired monomer isomer was dosed to the reaction mixture by two equal additions of 50 equiv at 0 and 3 h. 200 μL of a 140 mM DBU solution in dry NMP was added to start each polymerization reaction (corresponding to a 1:1 [OH]/[DBU] molar ratio), thus providing a starting concentration of 1 M in monomer and 0.02 M in OH groups (corresponding to 20, 10, 5, 3.33, 2.5, and 0.14 mM concentrations of BnOH, BDM, PET, diPET, Sucr, and PVA, respectively). After 3 h, the remaining 50 equiv of the monomer solution (2 mL) was added, followed by 800 μL of fresh NMP, so that the monomer concentration was maintained at 1 M, while the desired final [M]/[OH] molar ratio of 100 was reached. Indeed, the initial [M]/[OH] ratio was set to a DP_arm_ of 50, thus lower than the target value, in order to not exceed a monomer concentration of 1 M during the reaction.

Polymerization experiments were monitored over a 24 h period: aliquots (∼50 μL) were collected at predetermined time points and placed in glass vials each containing a NMP solution of acetic acid (3-fold excess to the estimated DBU concentration in the aliquot). If the additional monomer was added at intermediate time points, samples were collected prior to its addition. These samples were then dried under vacuum in a Genevac centrifugal evaporator (SP Scientifics, US) for 60 min at 30 °C, 1 mbar. The obtained pellets were dissolved in CDCl_3_ for ^1^H NMR analysis and characterized by triple-detection GPC/SEC using THF as the eluent. After 6 h, most of the crude reaction mixture (5 mL) was collected and added to a solution of acetic acid in NMP (3:1 ratio to DBU) to stop the polymerization. Excess NMP was removed by a Genevac centrifugal evaporator (1 mbar, RT), and the crude product was redissolved in 2 mL of DCM and precipitated in cold isoamyl alcohol (1:15 solvent/nonsolvent volume ratio). After centrifugation, the process was repeated using cold methanol as a nonsolvent. The final product was dried by means of a Genevac centrifugal evaporator (1 mbar, RT) to a fine white powder and characterized by ^1^H NMR and GPC/SEC in THF (or SLS analysis in THF).

^1^H NMR (CDCl_3_): the atom numbering is provided in Scheme 1B and representative spectra are in Supporting Information, Figure S1. *Linear* (“*1-armed*”). δ = 1.45–1.55 (terminal CH(C**H**_**3**_); ***c***), 1.55–1.70 (main chain CH(C**H**_**3**_); ***b***), 2.75–2.80 (CO**H**(terminal); ***e***), 4.30–4.45 (terminal C**H**(CH_3_); ***d***), 5.10–5.30 (C**H**(CH_3_) main chain; ***a***), 7.31–7.42 ppm (aromatic C**H** on BnOH ring; ***g***, ***h***, ***i***). *Linear* (“*2-armed*”). δ = 1.45–1.55 (terminal CH(C**H**_**3**_); ***c***), 1.55–1.70 (main chain CH(C**H**_**3**_); ***b***), 2.75–2.80 (terminal CO**H**; ***e***), 4.30–4.45 (terminal C**H**(CH_3_); ***d***), 5.10–5.30 (main chain C**H**(CH_3_); ***a***), 7.34 ppm (aromatic C**H** on BDM ring; ***k***). *4*-*Armed star*. δ = 1.45–1.55 (terminal C(H)C**H**_**3**_; ***c***), 1.55–1.70 (main chain CH(C**H**_**3**_); ***b***), 2.75–2.80 (terminal CO**H**; ***e***), 4.05–4.25 (methylene CC**H**_**2**_O; ***l***), 4.30–4.45 (terminal C**H**(CH_3_); ***d***), 5.10–5.30 ppm (main chain C**H**(CH_3_); ***a***). *6*-*Armed star*. δ = 1.45–1.55 (terminal C(H)C**H**_**3**_; ***c***), 1.55–1.70 (main chain CH(C**H**_**3**_); ***b***), 2.75–2.80 (terminal CO**H**; ***e***), 4.00–4.25 (CC**H**_**2**_O methylene; ***m***—only qualitatively detectable, possibly overlapping with ***n***), 4.30–4.45 (terminal C**H**(CH_3_); ***d***), 5.10–5.30 ppm (main chain C**H**(CH_3_); ***a***). *8-Armed star*. δ = 1.45–1.55 (terminal C(H)C**H**_**3**_; ***c***), 1.55–1.70 (main chain CH(C**H**_**3**_); ***b***), 2.75–2.80 (terminal CO**H**; ***e***), 4.30–4.45 (terminal C**H**(CH_3_); ***d***), 5.10–5.30 ppm (main chain C**H**(CH_3_); ***a***). *Comb*. δ = 1.45–1.55 (terminal C(H)C**H**_**3**_; ***c***), 1.55–1.70 (main chain CH(C**H**_**3**_); ***b***), 2.75–2.80 (terminal CO**H**; ***e***), 4.30–4.45 (terminal C**H**(CH_3_); ***d***), 5.10–5.30 ppm (main chain C**H**(CH_3_); ***a***).

## Results and Discussion

### Synthetic Procedures and Macromolecular Characterization

**Figure 1:**
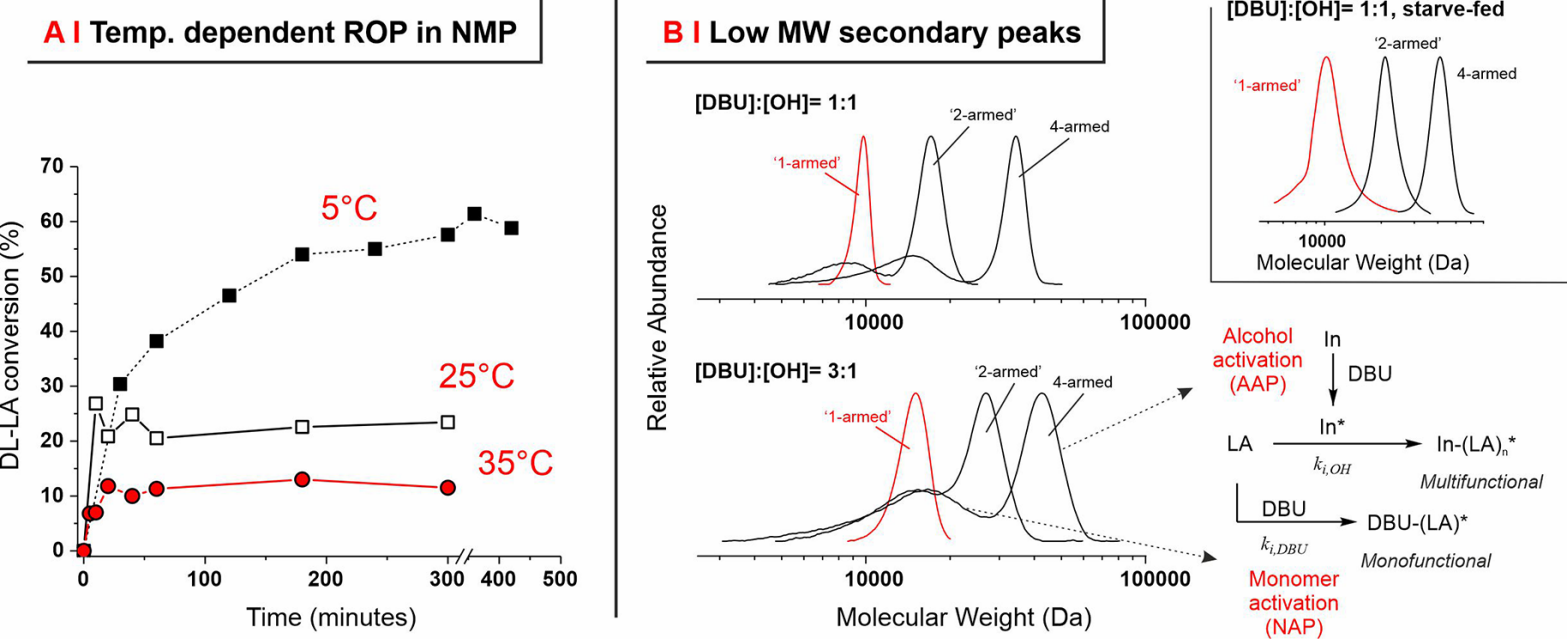
(A) Conversion of DL-LA in ROP conducted at 35, 25, and 5 °C and initiated by benzyl alcohol in NMP. [OH]/[DBU] = 1:1, [DL-LA] = 1 M, and theoretical DP = 100. (B) MW distributions obtained *via* triple-detection GPC/SEC in THF for DL-LA polymerization in NMP at 5 °C (theoretical DP = 100 and [DL-LA] = 1 M) initiated by BnOH (red curve, “1-armed”), BDM (“2-armed”, *i.e.*, linear with two OH terminal groups), and PET (4-armed star architecture). For all samples, the low-MW peaks are attributed to polymerization *via* the NAP mechanism and the high-MW peaks to polymerization *via* the AAP. In the inset, the GPC/SEC traces are obtained from corresponding experiments of starve-fed polymerization, where the low-MW peak is absent. In the bottom right scheme, “In” stands for the initiator.

ROPs are thermodynamically driven processes, and the equilibrium between growth and depolymerization typically changes with temperature; as previously mentioned, a polymerization temperature well below *T*_c_, or a high *T*_c_, is generally beneficial to increase monomer conversion. Polar solvents can be detrimental, because they may significantly decrease the *T*_c_ of a ROP process; this is the likely cause of the failure of TBD-catalyzed lactide ROP at 30 °C [copolymerization of L-LA with dimethylmorpholinedione, <10% conversion of both monomers after 4 days, and 1 M total initial monomer concentration], when conducted in polar NMP.^29^ In order to demonstrate this point, we have conducted the DL-LA polymerization in NMP at different temperatures (Figure 1A) and variable monomer concentrations (see Supporting Information, Figure S2), clearly showing that monomer conversion increases at lower temperatures; of note, the increase in NMP viscosity and the decrease in initiator solubility also cast a lower limit to the polymerization temperature, which here we have limited to 5 °C. Such results indicate that in a highly polar medium such as NMP, the monomer concentration at equilibrium ([M]_eq_) is higher than that in more popular solvents such as CHCl_3_ or dichloromethane, where under comparable conditions, completion can be achieved within 1 h at RT.^7,11^

However, when the low-temperature ROP in NMP was conducted with multifunctional initiators (2- and 4-armed; [I]/[M] = 1:100; 1 M [LA]), the corresponding MW distributions showed a secondary, lower-MW peak already present at a [DBU]/[OH] (catalyst to initiator) ratio = 1 and increasing with larger [DBU]/[OH]values (Figure 1B and Supporting Information, Table S1). This peak was not observed when using monofunctional initiators with comparable [DBU]/[OH] values, both in our low-temperature NMP experiments and in the literature RT dichloromethane studies.^11^ We hypothesize that this fraction of smaller polymers is composed of linear macromolecules generated through a NAP. Their possible presence would go essentially undetected when the AAP produces a linear polymer too, the only significant difference being one of the terminal groups; on the contrary, when using multifunctional initiators, the AAP yields much larger macromolecules, which would allow the NAP-produced smaller and linear polymers to be clearly separated in size (Figure 1B, scheme on the right). Incidentally, the use of multifunctional initiators may become useful in ROP, in order to determine the AAP/NAP performance of a given polymerization system.

Both the [OH]/[DBU] and the [LA]/[DBU] ratios influence the occurrence of the NAP.^11^ A higher [OH]/[DBU] ratio would reduce the NAP, but a lower DBU content would also significantly reduce the initiation (*k*_i,OH_; Figure 1B, right) and propagation, which are already relatively slow in NMP at low temperature. Conversely, provided a sufficiently fast AAP rate, the equilibrium of propagation can be shifted toward the products by decreasing the [LA]/[DBU] molar ratio, which ensures a high propagation rate and a minimum amount of the monomer available for alternative initiations. The starve-fed, also known as the monomer-starved, approach employs multiple additions of the monomer during the polymerization; this allows us to significantly reduce the maximum possible [LA]/[DBU] ratio at any given polymerization time while actually using large overall amounts of the monomer. Following this approach, in all polymerizations, we have dosed the monomer in two equal, stacked additions: 50 equiv at 0 h and 50 equiv at 3 h; the [OH]/[DBU] ratio was maintained at 1 throughout the process.

**Figure 2:**
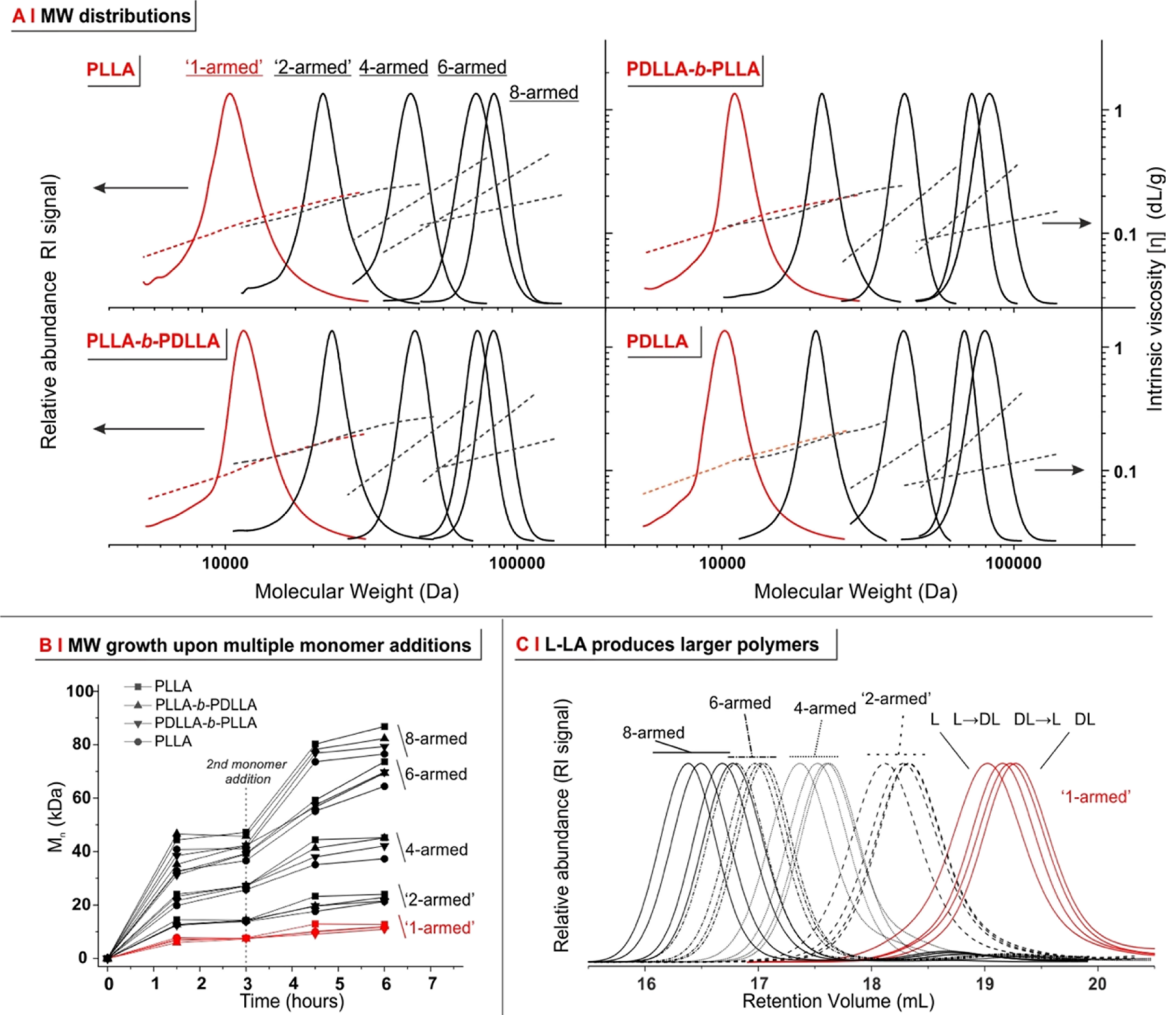
(A) MW distributions and Mark–Houwink plots for linear/star PLAs with different stereochemistry (PLLA, PLLA-*b*-PDLLA, PDLLA-*b*-PLLA, and PDLLA; for the complete GPC traces, see Supporting Information, Figure S3). 1- and 2-armed (both linear) polymers show the same dependency of [η] on MW, whereas for comparable MWs, [η] decreases with increasing branching. Please note that the MW determination of comb samples through GPC/SEC was inconclusive, exceeding the column size-exclusion limit (see Supporting Information, Figure S4), and therefore, it is limited to the weight-average MW () obtained through SLS in the batch mode (see Supporting Information, Table S5, with corresponding Zimm plots in Figure S4). Of note, GPC/SEC can provide the Mark–Houwink *a* parameter and the hydrodynamic radius *R*_H_ of these polymers (see Figure 4) since this analysis does not necessarily require polymer fractionation. (B) Growth in molar mass (number-average MW, ) over the course of a 2-addition (monomer-starved), 6 h polymerization for the linear (1- or 2-armed) and star (4-, 6-, and 8-armed) PLAs with variable tacticity, as monitored by GPC/SEC. The increase in molar mass after the second addition is, incidentally, an indirect indication of the living character of the polymerization. The complete data (conversion and *M*_*n*_ from NMR and *M*_*n*_, *M*_*nw*_, and Đ from GPC/SEC) for all starve-fed polymerization experiments are presented in Supporting Information, Table S2. (C) GPC/SEC chromatograms (normalized RI *vs* retention volume) of 1-, 2-, 4-, 6-, and 8-armed PLAs obtained under monomer-starved conditions with the feed composed of L-LA only, DL-LA only, or an alternated addition of the two isomers.

In this way, narrow and monomodal MW distributions were always obtained (Figure 2A and Table 1), provided that a high monomer conversion was attained before the second addition (Figure 2B).

**Table 1:** Macromolecular Characterization for Multiarmed PLAs with Diverse Tacticity and Branching

sample^a^	arms^b^	conv.^c^ (mol %)	d (kDa)	e (kDa)	e (kDa)	Đ^e^	[η]^e^ (dL/g)
*L*_(PLLA)	1	88	12.7	12.7	14.4	1.13	0.151
*L*_(PLLA)_2_	2	88	24.1	24.0	25.5	1.05	0.169
*S*_(PLLA)_4_	4	86	49.5	44.1	47.8	1.08	0.173
*S*_(PLLA)_6_	6	89	76.9	74.6	76.1	1.02	0.197
*S*_(PLLA)_8_	8	75	92.4	86.8	88.9	1.02	0.155
*C*_(PLLA)_140_	∼140	78	1572		1913^f^	“1.21”	0.311
*L*_(PLLA-*b*-PDLLA)	1	81	11.5	11.9	12.3	1.05	0.134
*L*_(PLLA-*b*-PDLLA)_2_	2	82	23.5	22.9	24.0	1.05	0.178
*S*_(PLLA-*b*-PDLLA)_4_	4	79	45.4	43.2	47.2	1.09	0.145
*S*_(PLLA-*b*-PDLLA)_6_	6	76	65.7	69.4	74.0	1.07	0.180
*S*_(PLLA-*b*-PDLLA)_8_	8	77	81.8	82.3	82.8	1.01	0.135
*C*_(PLLA-*b*-PDLLA)_140_	∼140	69	1391		2033^f^	“*1.46*”	0.265
*L*_(PDLLA-*b*-PLLA)	1	79	11.4	11.8	11.4	1.03	0.136
*L*_(PDLLA-*b*-PLLA)_2_	2	80	22.9	21.5	23.7	1.10	0.172
*S*_(PDLLA-*b*-PLLA)_4_	4	78	45.1	42.1	46.5	1.07	0.154
*S*_(PDLLA-*b*-PLLA)_6_	6	77	66.9	69.7	77.3	1.11	0.172
*S*_(PDLLA-*b*-PLLA)_8_	8	73	91.2	79.3	89.0	1.12	0.115
*C*_(PDLLA-*b*-PLLA)_140_	∼140	73	1472		1673^f^	“*1.13*”	0.306
*L*_(PDLLA)	1	84	12.1	10.8	11.7	1.10	0.129
*L*_(PDLLA)_2_	2	83	23.9	22.9	24.5	1.07	0.163
*S*_(PDLLA)_4_	4	82	47.2	38.3	42.5	1.11	0.135
*S*_(PDLLA)_6_	6	79	68.3	64.4	70.6	1.10	0.15
*S*_(PDLLA)_8_	8	76	87.4	76.5	81.1	1.06	0.103
*C*_(PDLLA)_140_	∼140	67	1351		1229^f^	“*1.00*”	0.262

a *L* stands for linear, *S* for star, and *C* for comb. [monomer] = 1 M monomer, polymerized in NMP at 5 °C for 6 h, with a theoretical DP_arm_ = 100 and using DBU in an equimolar ratio to the OH groups: (*i.e.*, [M]/[OH]/[DBU] = 100:1:1).

b Theoretical number of arms = functionality of the initiator. Since in no polymer did we observe the ^1^H NMR resonances of unreacted alcohols (at, *e.g.*, δ = 3.8 ppm for PET), the actual and theoretical arm numbers were assumed to be identical.

c DL-LA and L-LA were added to the reaction mixture by staggered additions in a starve-fed (monomer-starved) polymerization with a DL/L molar ratio of 1:1 (0.5 equiv at 0 h and 0.5 equiv at 3 h). The conversion is expressed as the consumed monomer at a given time point, as detected by ^1^H NMR in CHCl_3_ (methine signal, ratio of the monomer and polymer resonances).

d Calculated from the monomer conversion, as measured in situ by ^1^H NMR in CHCl_3_.

e MW averages, dispersity (Đ), and intrinsic viscosity ([η]) are obtained *via* triple-detection GPC/SEC in THF.

f of comb polymers derives from batch-mode SLS [see Supporting Information, Table S5, which also provides radii of gyration (*R*_g_)] since their large size hampers GPC/SEC; their Đ values (in italics) are obtained using NMR  values.

It is of note that not only linear (*L* prefix) but also star polymers (*S* prefix) were characterized by very low dispersity values, typically <1.1; this demonstrates the versatility of the polymerization method, which allows for very well-defined structures also for a variety of (very polar) initiators. For the comb structures obtained using PVA as an initiator (*C* prefix), GPC/SEC analysis could only provide an estimate of their average intrinsic viscosity, due to their excessively large MW, as assessed *via* SLS in THF.

Another noteworthy point is that independently of the macromolecular architecture, the use of L-LA was always associated with a 10–20% larger MW (Figure 2C); since L-LA and DL-LA were purified in an identical manner and have the same chemical structure, we are inclined to ascribe this effect to a better availability of PLLA terminal groups during polymerization. In order to further confirm the stereochemistry of the branches, we have followed a literature protocol for the homonuclear decoupling of methine and methyl resonances in the ^1^H NMR spectra (compare Figure 4A,B).^24,25^ The resulting improved resolution of the methine resonance allows the quantitative assessment of individual tetrads and hexads, which are named according to their internal succession of racemic (*r*) and meso (*m*) diads; we have here determined the probability P*r* for a diad to be *r* (numerical data reported in Supporting Information, Table S4), which numerically would range between 0 for fully isotactic PLLA and 0.5 for atactic PDLLA; please note that PDLLA contains isotactic diads arising from the monomer itself.^30^

**Figure 3:**
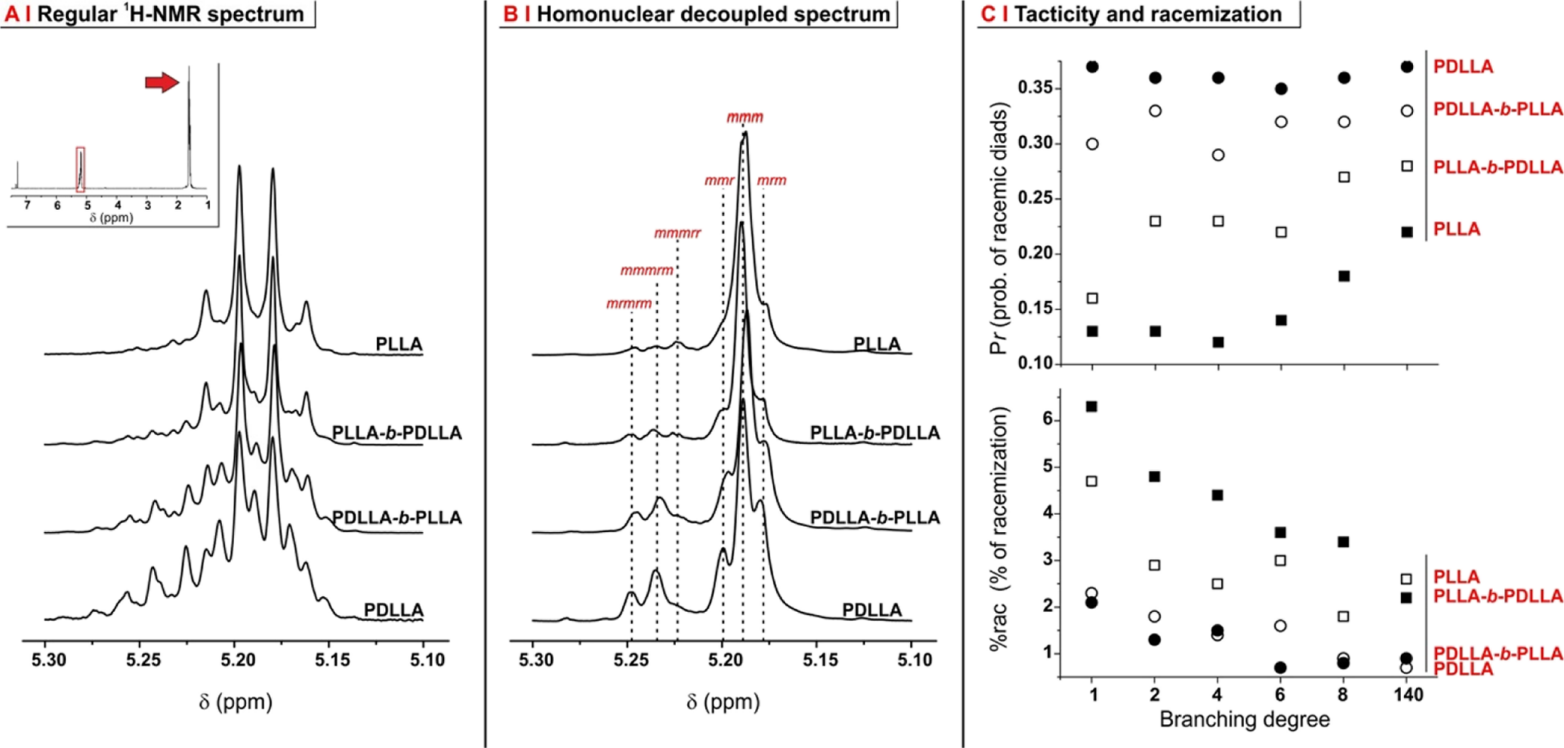
(A) Methine region (5.1–5.3 ppm) of 2-armed PLAs in ^1^H NMR spectra in CDCl_3_; the inset shows the complete spectrum; and the arrow indicates the methyl resonance, which is irradiated to decouple it from methine. (B) Methine region after homonuclear decoupling, which allows for a clearer assessment of tetrads and hexads (in red). Please note that elsewhere, *m* diads may be referred to as isotactic (*i*) and *r* diads as syndiotactic (*s*). (C) Probability of occurrence of an *r* diad (top) and degree of racemization (bottom) as a function of the branching degree for PLLA, PDLLA, and their block copolymers.

The low-temperature DBU-catalyzed ROP in NMP shows a predominantly isotactic character (Figure 3C, top), in line with the other reports for DBU- and TBD-catalyzed ROPs:^7,31^ for linear and low-branching polymers, P*r* values of 0.13–0.16 are recorded for PLLAs and 0.35 for PDLLAs. We are inclined to ascribe the P*r* values of the linear polymers to DBU-induced epimerization, slightly higher than most values in the literature: epimerization requires an anionic intermediate, more stable in NMP than, for example, in apolar organic solvents such as toluene or in bulk. The P*r* values for PLLA blocks further increase in the more densely branched structures, such as 8-armed stars and combs. Since the latter polymers also have the highest local DBU concentration during polymerization, this trend may further indicate P*r* to be specifically affected by epimerization.

Importantly, the location of the PLLA block seems to be important; for all degrees of branching, the constructs where PLLA is close to the initiator (= L-LA polymerized first, PLLA-*b*-PDLLA) show a higher degree of isotacticity than those where L-LA is in the second block (= DL-LA polymerized first, PDLLA-*b*-PLLA). In this second case, two factors contribute to the lower stereoregularity: (A) when L-LA is added, some residual DL-LA “spillover” is still present (in most cases, 2–6% of the original feed; see the conversion data reported in Supporting Information, Table S2); this will introduce DL-LA defects in the PLLA block and thus reduce its stereotactic order. (B) The second block is not only less stereoregular but also shorter: as an average, about 70% of the second batch of the monomer was consumed (at the end of the polymerization, 6 h; see the conversion data reported in Supporting Information, Table S2).

The resonance of *mmmrr* hexads (5.22 ppm) was then used as a quantitative measure of racemization reactions: these hexads are generally absent in PLAs produced from either L-LA or DL-LA since they formally derive from *meso* lactide, but they can appear upon racemization obtained through transesterification.^26,32^ The parameter %rac obtained from this resonance indicates a low percentage of racemization for all polymers (<6%), especially when compared to PLAs obtained *via* bulk polymerizations.^33^ Interestingly, racemization appears to decrease with increasing branching; since the latter reduces intermolecular entanglements (see the next paragraph), it seems logical to hypothesize that this form of topological interactions is the main source of racemization. Further, racemization apparently increased with the presence of stereoregular blocks: at any branching degree, the %rac of PLLA-*b*-PDLLA (= L-LA polymerized first, more stereoregular PLLA block) was always similar to that of PLLAs, higher than that of PDLLA-*b*-PLLA (= DL-LA polymerized first, less stereoregular PLLA), and much higher than that of PDLLA.

**Figure 4:**
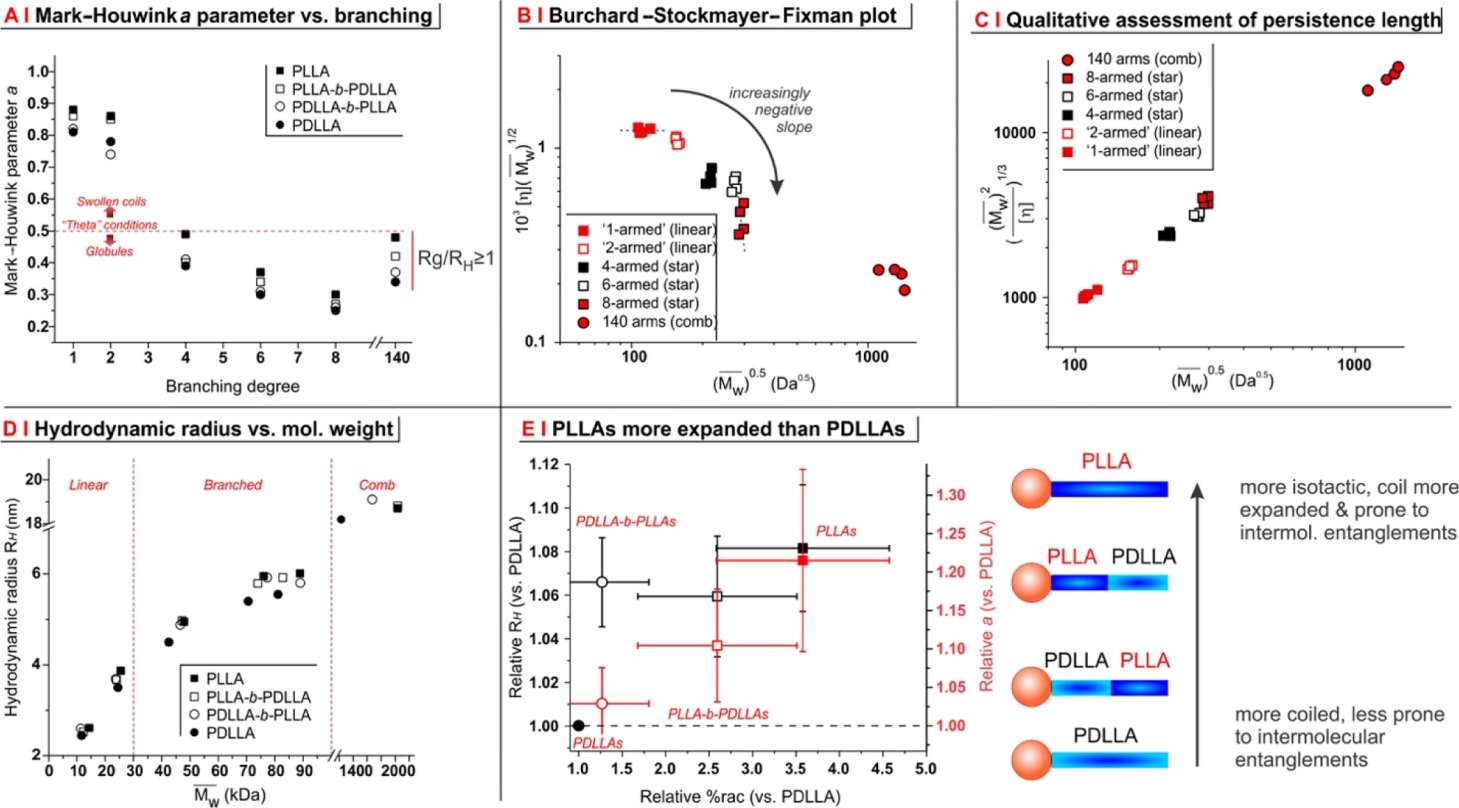
(A) Mark–Houwink (conformational) parameter *a vs* branching degree; all star PLAs are compact, rather globular structures (0.2 ≥ *a* ≥ 0.5), while comb PLAs are characterized by an *R*_g_/*R*_H_ ratio (*R*_g_ from SLS experiments and *R*_H_ calculated as in C) above 1. (B) Plot of *vs* (from the Stockmayer–Fixman theory and the derived Burchard–Stockmayer–Fixman expression), where *K*_θ_ is the Mark–Houwink *K* coefficient under Θ conditions, Φ is the Flory–Fox parameter, and *B* is a parameter that acquires positive or negative values, respectively, above and below the Θ temperature. (C) The slope of the plot of *vs* is inversely proportional to the persistence length of a polymer chain, derived from the complete Bohdanecky relation, (39) where *A*_0_ and *B*_0_ are adjustable coefficients, *M*_L_ and *L*_P_ are, respectively, the mass per unit length and the persistence length of the polymer, and Φ is the Flory–Fox parameter. (D) The weight-average viscosimetric hydrodynamic radius is calculated as , as a direct derivation from Einstein’s viscosity equation.^40^ The hydrodynamic radius grows roughly as the cubic root of , that is, the density of the macromolecular coils is essentially independent of branching and stereochemistry. Please note that most polymers are <10 nm in size; thus, the radius of gyration (*R*_g_) was not considered due to the unreliability of Rayleigh scattering of such small coils when using red-light sources. (E) The values of %rac (horizontal axis), *R*_H_ (left vertical axis, black), and Mark–Houwink parameter *a* (right vertical axis, red) are normalized against PDLLA and averaged over the different degrees of branching. Although most differences are not statistically relevant, a trend toward larger dimensions, more expanded coils, and a higher racemization is apparent when moving from PDLLAs to PLLAs.

We then assessed the effect of branching and stereoregularity on hydrodynamic properties, first focusing on the Mark–Houwink parameter *a*. An *a* value of 0.5 indicates coils under theta (θ) conditions; lower values—typically seen with increasing branching (see, *e.g.*, with propylene sulfide homo-^34^ and copolymers^35^)—reflect increasing contraction and lower propensity to entanglement; this trend was also recorded for PLAs (Figure 4A), which confirms their progressively lower tendency to entangle.

The values of the Mark–Houwink parameter *a* also suggest the star polymers to be progressively below θ conditions; we have qualitatively confirmed this in a Burchard–Stockmayer–Fixman plot, where each group of differently branched polymers appears to show an increasingly negative slope (= larger distance from θ conditions^36^) with increasing branching (Figure 4B). Comb polymers appear closer to θ conditions than their degree of branching would suggest (higher value of the parameter *a* and a lower slope in the Burchard–Stockmayer–Fixman plot), which is likely a consequence of the PVA chain being stretched due to the high density of branching chains, as also suggested by a shape factor of (*R*_g_/*R*_H_) ≥ 1.

Importantly, the differential expansion of the coils for the various macromolecules does not appear to be directly related to a significantly different chain rigidity. For a given macromolecular structure, the slope of a *versus* graph is inversely related to its persistence length; all polymers produced in this study nicely fit with a single slope (Figure 4C), suggesting a negligible effect of branching. This is likely due to the long PLA chains (100 monomeric units per arm), which overcome the single branching point; this would not be the case for shorter chains, and indeed, we have recently showed that short-armed stars (polysulfides, 10–30 units per arm) have a larger persistence length than linear analogues.^35^

Another point is noteworthy; chain dimensions increase with MW, but for any branching except combs, the size of PLLAs is always the largest and that of PDLLAs the smallest (black squares and black circles in Figure 4D), and this difference is larger than what is expected on the basis of the modest differences in MW. In summary, the more stereoregular chains—that is, PLLAs and, to a lower extent, PLLA-*b*-PDLLAs—appear to be slightly more reactive during synthesis, more prone to racemization through intermolecular interactions, and more expanded in solution than their less stereoregular counterparts (PDLLAs and PDLLA-*b*-PLLAs); this behavior is observed for all linear and star polymers and indeed becomes apparent if data are normalized against PDLLAs and averaged through the different degrees of branching (Figure 4E). Interestingly, the second virial coefficient (A2) recorded *via* SLS on comb polymers in THF increases in the order PLLA ∼ PLLA-*b*-PDLLA < PDLLA-*b*-PLLA ≪ PDLLA (see Supporting Information, Table S5); this tallies with the racemization data and further confirms that the latter two backbones are the least prone to intermolecular interactions. All these effects may be tentatively linked to the conformation of the stereoregular PLLA segments, which, for example, are able to adopt a left-handed helical conformation in solution;^37,38^ on the contrary, the always randomly coiled atactic PDLLA chains may offer a higher steric hindrance to monomer addition and adopt a more compact conformation, which is also less prone to intermolecular entanglements.

### Bulk Properties

**Figure 5:**
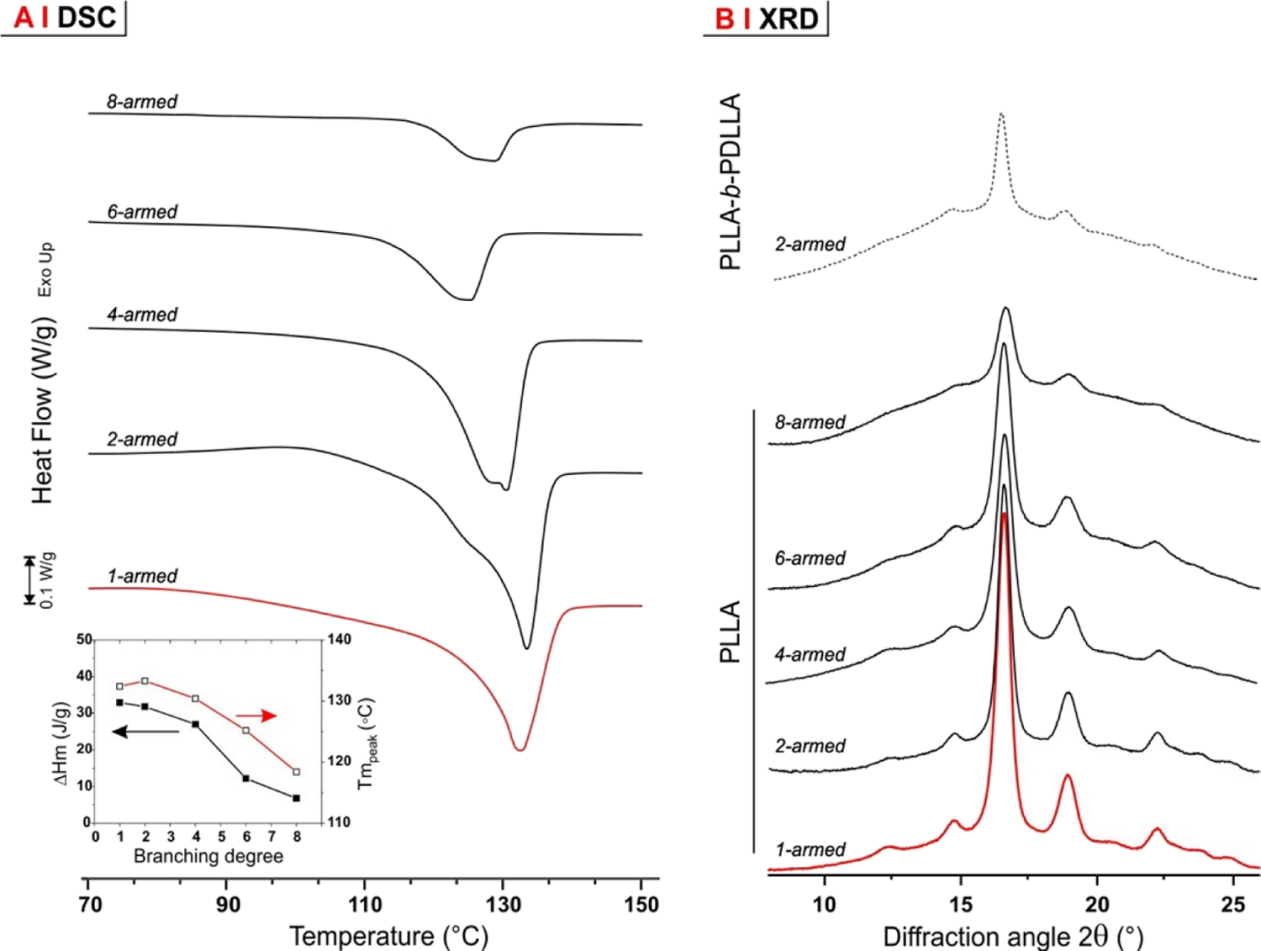
(A) Melting region in first-heating DSC scans for PLLAs with different degrees of branching (C_PLLA excluded since it did not show any melting endotherm). (B) Powder XRD diffraction patterns of all semicrystalline polymers.

Neither the degree of branching nor stereoregularity showed any significant effect on the glass transition of any polymer (*T*_g_ always between 47 and 55 °C; see Supporting Information, Table S6) but strongly affected the crystallizability of PLLA. DSC analysis showed that, as expected, homopolymeric PLLAs became less crystalline with increasing branching (Figure 5A) due to the lower likelihood of intermolecular assemblies; on the contrary, melting endotherms were completely absent from all PDLLA-*b*-PLLAs and also from most PLLA-*b*-PDLLAs; however, among the latter, the polymer with the longest unbranched sequence of L-LA units—that is, the 2-armed PLLA-*b*-PDLLA—did show a low (6%) but detectable crystallinity, as confirmed also by XRD analysis (Figure 5B). This is an indication that, as we previously observed in solution, the intermediate character of PLLA-*b*-PDLLAs is between those of the more ordered PLLAs and the less ordered PDLLA-*b*-PLLAs, and PDLLAs also appear in bulk. In order to confirm this, we have further focused on a 6-armed star as a single degree of branching and assessed any possible rheological/mechanical difference related to the stereochemistry of its branches.

**Figure 6:**
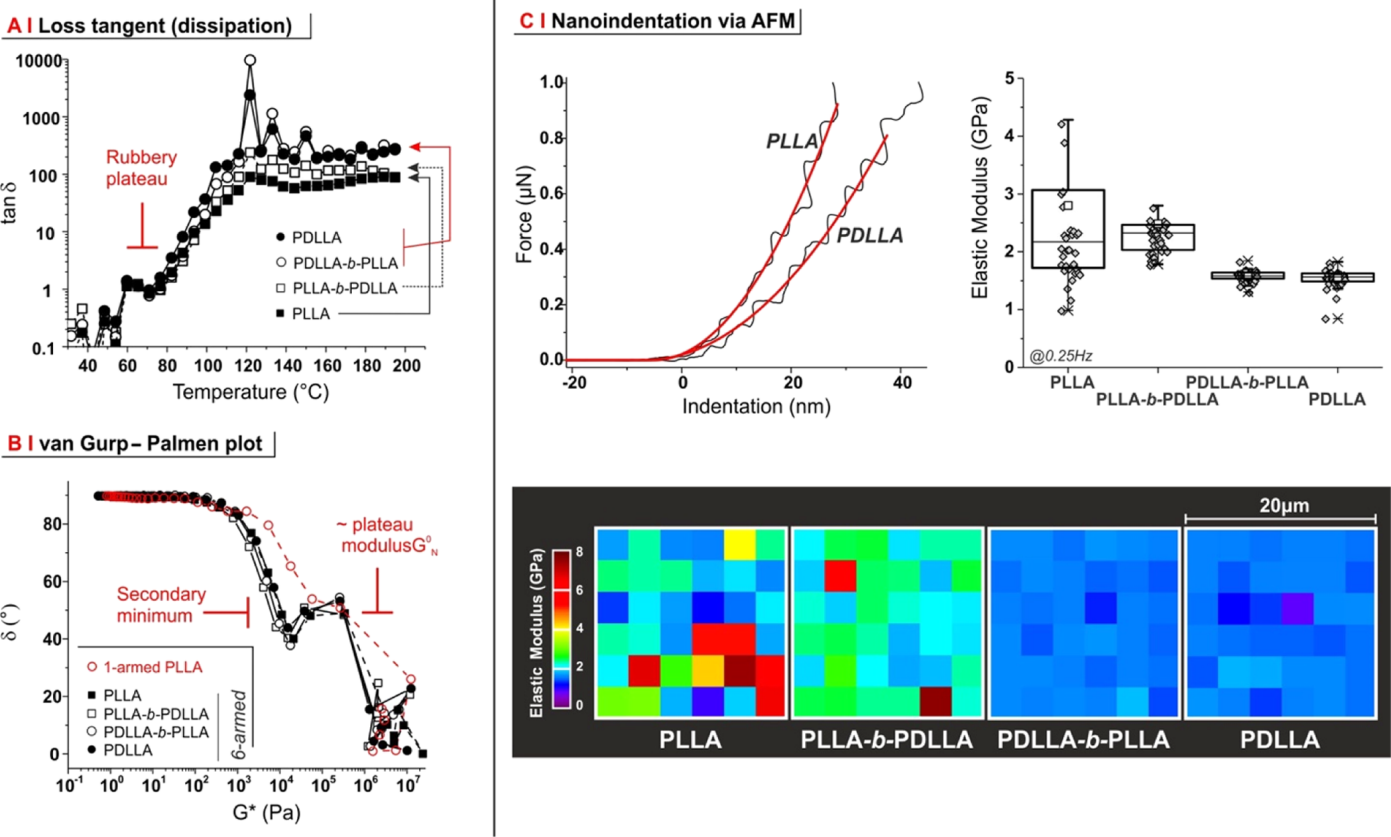
(A) Dependence of tangent δ (dissipation/loss factor = *G*″/*G*′) on temperature for 6-armed PLAs; the end of the rubbery plateau is easily recognizable (red bar), while the tan δ peak typically expected in correspondence to the glass transition was not observed. Please also note that the measurements were performed *via* a relatively rapid cooling from the melt (5 °C/min), which prevented significant crystallization. The corresponding *G*′ and *G*″ curves are provided in Supporting Information, Figure S6B. (B) vGP plot for the 6-armed PLAs (black symbols) and L_(PLLA) (1-armed, linear PLLA). All polymers exhibited a primary minimum (branch relaxation) in correspondence of the plateau modulus, but only branched polymers showed a secondary minimum (whole macromolecule relaxation). (C) From AFM nanoindentation (top left), it is possible to calculate the (surface) elastic modulus of 6-armed PLA films (top right); note that the indentation rate—here, 0.25 Hz—is a crucial parameter; at higher indentation rates, no trend can be observed (see Supporting Information, Figure S7). Of note, PLLA and, to a lesser degree, PLLA-*b*-PDLLA samples showed a higher heterogeneity in the surface modulus, as highlighted in force maps taken at 0.25 Hz (bottom).

In the melt, all 6-armed star polymers showed a very similar temperature dependence of complex viscosity (η*), with slightly lower η* values for PDLLA (see Supporting Information, Figure S6A); correspondingly, PDLLA also showed lower values of *G*′ and *G*″ (see Supporting Information, Figure S6B). The analysis of tan δ as a measure of the dissipation characteristics of the various polymers was more informative (Figure 6A): in the melt, the “fully amorphous” polymers (PDLLA and PDLLA-*b*-PLLA) were indistinguishable and showed the highest dissipation; lower tan δ values were recorded for PLLA-*b*-PDLLA and even lower for PLLA, which tallies with their previously hypothesized higher propensity toward intermolecular entanglements.

An important confirmation analysis was performed with can be obtained through the help of a van Gurp–Palmen plot (vGP plot, *i.e.*, graph of the loss angle δ *vs* the complex shear modulus G*^41^). Although more typically employed for data from frequency sweeps,^42−44^ the time–temperature superposition principle^41^ allows the use of the vGP plot for temperature-dependent measurements also, as in this case. It has been demonstrated that in a series of homologous polymers, the shape of a vGP plot depends only on MW and its dispersity^43^ and on the degree of branching^44^ but not on tacticity.^43^ Indeed, the 6-armed polymers almost perfectly overlapped (Figure 6B), which confirms the tacticity of their arms as the only significant difference among them. Of note, the vGP plots for these star polymers showed a first minimum at a G* value of about 10 kPa and a deeper minimum roughly in correspondence to the plateau modulus *G*_N_^0^; this is an additional confirmation of their branched nature since two minima are commonly observed in branched macromolecules but not in their linear analogues,^42,45^ and indeed, a linear, 1-armed PLLA provided a considerably different vGP plot, devoid of the first minimum (red symbols in Figure 6B). According to an interpretation used for comb polymers,^42^ the second minimum is tentatively associated to the relaxation of individual arms or linear chains, while the first is related to the branching area of the polymer.

The differences seen in the melt between the stereochemically different 6-armed polymers were also recorded in the solid state. Force maps obtained *via* atomic force microscopy (AFM) in the nanoindentation mode showed that PLLA and PLLA-*b*-PDLLA had a higher average hardness and a larger surface mechanical heterogeneity than their “fully amorphous” counterparts (Figure 6C); these effects are likely a consequence of the surface exposure of (hard) crystalline domains, which are absent in both PDLLA-*b*-PLLA and PDLLA.

In summary, bulk properties substantially mirrored the results obtained in solution: PLLA-*b*-PDLLA and PDLLA-*b*-PLLA behave differently, with the second being very similar to PDLLA and the first intermediate between PDLLA and PLLA. In particular, PLLA-*b*-PDLLA shows clearer signs of intermolecular interactions, for example, with a higher modulus in nanoindentation, a lower tan δ value, and—in one case—a *T*_m_. Due to its central position, the PLLA block in PDLLA-*b*-PLLA is less hindered by the branching point than it is in PLLA-*b*-PDLLA; therefore, the different behavior should be ascribed—as previously seen for solution properties—to the more ordered conformation of the PLLA block in PLLA-*b*-PDLLA.

### Hydrolytic Degradation

**Figure 7:**
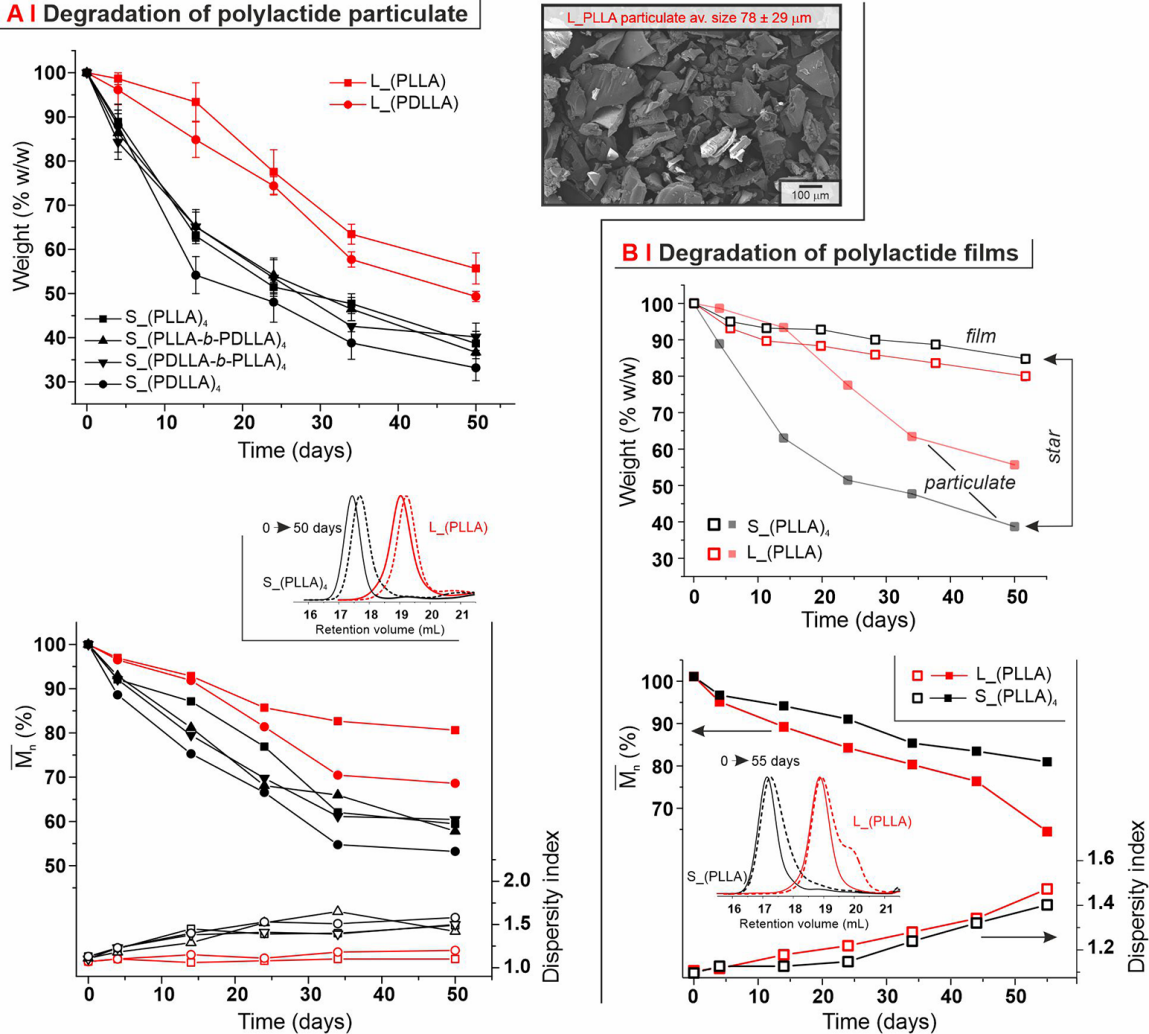
(A) Erosion profile (top) of PLAs in a particulate morphology (SEM image, right) and MW evolution (bottom) for linear (red symbols) and 4-armed star PLAs (black symbols) + during hydrolysis in PBS at 37 °C. Please note that the bottom graph reports number-average MW () data as a percentage of its value at day 0 (left axis; full symbols, higher part of the graph), and dispersity index (right axis; empty symbols, lower part of the graph) from triple-detection GPC/SEC measurements in THF; four representative curves are presented in the inset. (B) Erosion profile (top) and MW evolution (bottom) for linear (red squares) and 4-armed (black squares) PLLA in the form of films. The particulate erosion profile of the two polymers is presented in the top graph to highlight the profound effect of the morphology on the degradation above all of the branched polymers.

It is worth reminding that linear polyesters are well-known to undergo degradation predominantly through a bulk hydrolysis mechanism.^46^ Here, we have first compared the degradation profile of linear and 4-armed star PLAs in a particulate morphology (inset in Figure 7A); both mass loss and MW data (top and bottom graphs in Figure 7A, respectively) show that tacticity had a negligible influence on the degradation kinetics, whereas branching clearly accelerated it. Very interestingly, when the same polymers (linear and 4-armed star PLAs) were produced as films, the order of degradation inverted: the branched polymer underwent hydrolysis at a much slower pace than its linear analogue, which tallies with a previous report from us [films of Sn(Oct)_2_ produced linear and 4- or 6-armed star PDLLA].^5^ This evidence points toward a surface-dominated degradation mechanism for the branched polymers, which are more stable than the linear counterparts with low surface/volume ratios (such as in films) and less stable when the amount of the water-exposed surface is maximized.

## Conclusions

NMP is an advantageous solvent for the preparation of complex polymer architectures, for example, because of its ability to solubilize very polar initiators, such as polyols. Despite some negative literature reports, lactide ROP can take place in NMP; it proceeds under very mild conditions and, if the polymerization temperature is appropriately low and monomers are starve-fed, it affords very well-defined (low MW dispersity) structures, even at a branching of up to 140 branches per molecule.

This polymerization method opens the way to a very fine and—to our knowledge—unprecedented level of topological control for biodegradable polyesters. In particular, it is a very attractive perspective to be able to exert full control over even massive degrees of branching, while obtaining very low MW dispersity and—due to the low temperature—minimizing parasite reactions, including racemization.

The technological applications are numerous; for example, we have here demonstrated that branching significantly increases the surface-erosion component during PLA degradation, which may be employed to significantly accelerate the degradation of a material upon shredding. The availability of finely controlled branched structures will then allow for a precise tailoring of their transport and mechanical properties.

It is also noteworthy that this mechanism remains an equilibrium ROP; as such, the *one-pot* synthesis of multiblock structures is possible but also somehow limited by the monomer “spillover”: a sizeable amount of the first monomer will still present (and in equilibrium with the growing chain) when a second is added and will then give rise to defects in the second block. To demonstrate this, we have used tacticity as a sensitive tool to probe the purity of polymer blocks, showing that in PLLA-*b*-PDLLA (LLA polymerized first), the PLLA block is more ordered than in PDLLA-*b*-PLLA (LLA polymerized last), independent of the degree of branching. Correspondingly, the two apparently identical block copolymers differ in both solution and bulk behaviors: while PLLA-*b*-PDLLA is an intermediate between PLLA and PDLLA, PDLLA-*b*-PLLA is barely distinguishable from PDLLA.

## Supporting Information Available

Additional synthesis and characterization data including complete GPC curves, numerical tables for monomer conversion at different time points, and d*n*/d*c* data (PDF)
