# Supplementary material for: Versatile Preparation of Branched Polylactides by Low-Temperature, Organocatalytic Ring-Opening Polymerization in N-Methylpyrrolidone and Their Surface Degradation Behavior
Source: Macromolecules. 2021 Oct 7;54(20):9482–95. doi: 10.1021/acs.macromol.1c01503 (PMC8552446; doi:10.1021/acs.macromol.1c01503)
Supplement: Supplementary file 1 — ma1c01503_si_001.pdf [file ma1c01503_si_001.pdf]

A versatile preparation for branched polylactides; low-temperature, organocatalytic ring-opening polymerization in NMP permits synthesis of highly branched architectures with surface-dominated degradation properties

Supporting Information

Giulia Scoponi[1,2,¶], Nora Francini[3,¶], Veronica Paradiso[4], Roberto Donno[3], Arianna Gennari[3], Richard d'Arcy[3, §], Carmine Capacchione[4], Athanassia Athanassiou[1], Nicola Tirelli[3,5]*

[1] Smart Materials, Istituto Italiano di Tecnologia, via Morego 30, 16163 Genova, Italy.

[2] DIBRIS, University of Genova, Via Opera Pia 13, Genova 16145, Italy

[3] Laboratory of Polymers and Biomaterials, Istituto Italiano di Tecnologia, via Morego 30, 16163 Genoa, Italy.

[4] Department of Chemistry and Biology "Adolfo Zambelli", University of Salerno, Via Giovanni Paolo II 132 , 84084 Fisciano, Italy

[5] School of Health Sciences, University of Manchester, Oxford Road, Manchester M13 9PL, United Kingdom.

S1. ^{1}H-NMR spectra

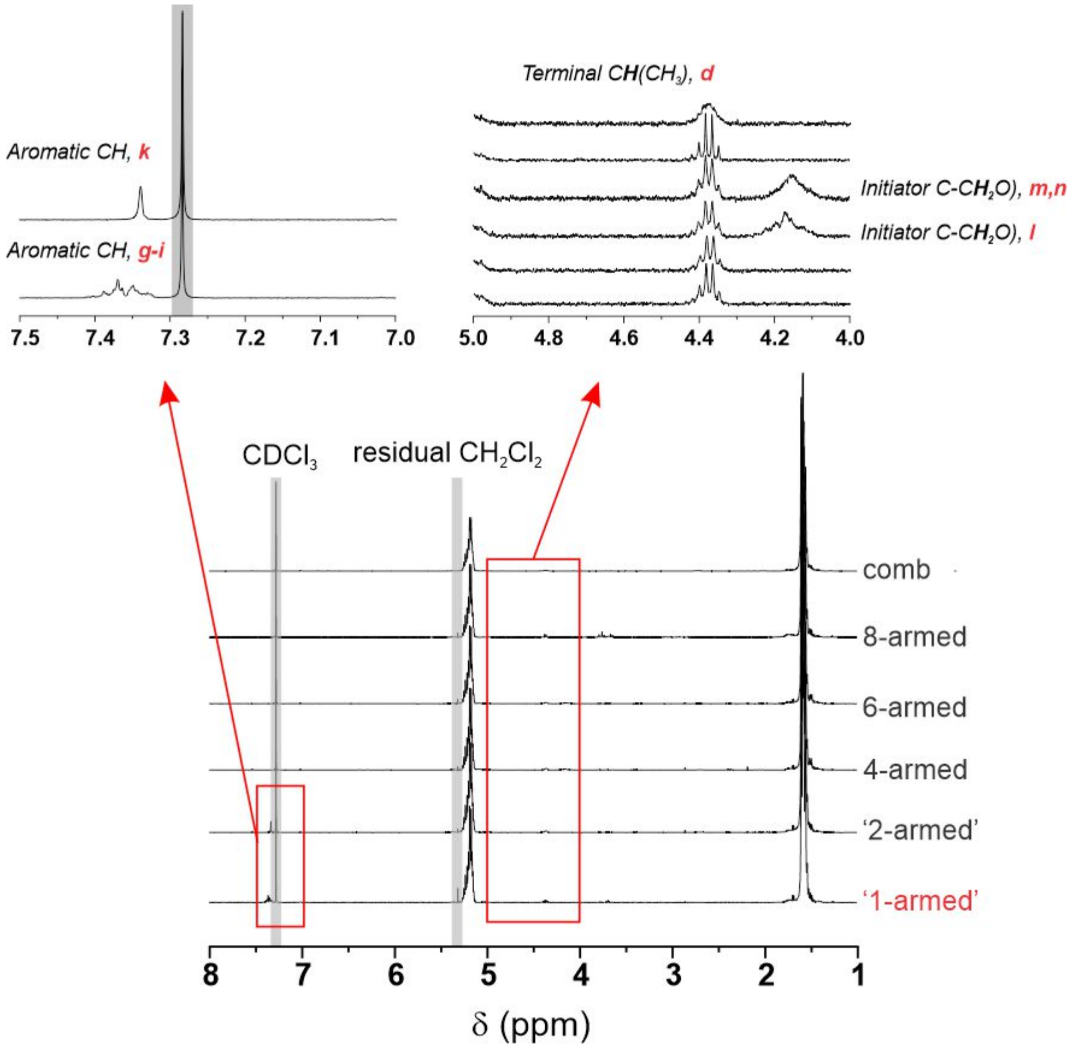

Figure S1. Representative ^{1}H-NMR spectra for PDLLA of variable degree of branching: the magnified areas show resonances of terminal groups, which can be used to calculate the number average degree of LA polymerization. Of note, in the 8-armed polymers it is impossible to identify any of the sucrose signals (note the absence of proton numbering for sucrose in Scheme 1B).

However, in those spectra it is still possible to identify the methine groups in the last repeating units, and the ratio between their resonance and that of the main chain protons is rather constant for all degrees of branching: in all polymers the number average degree of polymerization of the branches is always 80-85 units; for sucrose it is slightly less 80. This tallies with the monomer conversion (80% equates to around 80 monomers per OH). Assuming the branches to be all equally long, the Mn from GPC would correspond to 7.5 branches.

Clearly, they are unlikely to be all long the same, but all in all they have to compensate each other, therefore it seems reasonable to assume that more or less these polymers do have about 7-8 branches.

S2. Optimization of the synthetic conditions

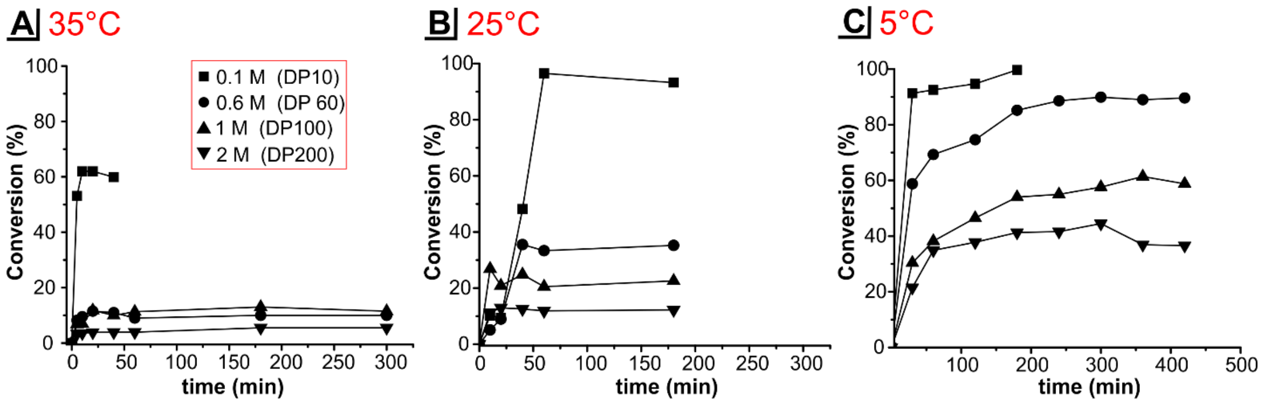

Figure S2. Conversion of DL-LA in the polymerization initiated by benzyl alcohol in NMP at 35, 25, and 5 °C. [OH]:[DBU] = 1:1; concentration of monomer and corresponding values of theoretical degree of polymerization are reported in the legend.

Table S1. $\overline{M_n}$ and dispersities of 1-, 2- and 4-armed PDLLA after 3 hours of polymerization in NMP. [LA] = 1 M, [LA]:[OH] = 100, T = 5 °C.

#arms	[DBU]:[OH]	Conversion (%)[a]	$\overline{M_n}$ GPC (kDa)[b]	Đ[b]	A (%)[c]
1	1:1	61	9.5	1.01	n.d.
2	1:1	68	15.6	1.12	18
4	1:1	64	35.2	1.23	19
1	3:1	92	14.5	1.09	n.d.
2	3:1	98	19.2	1.26	30
4	3:1	98	39.8	1.41	33

[a] Measured by ^{1}H-NMR after 3 hours, by comparing the methine signal of the residual monomer and of the polymer after quenching the polymerization with acetic acid, removal of the solvent and re-dissolution in $CDCl_3$.
[b] Measured by triple detection GPC in THF using. In the case of #arms >1, data correspond to average MW calculated from the obtained bimodal distribution.
[c] Relative contribution of the lower MW component, as calculated from the deconvolution of the RI signal curve.

S3. Complete reaction data for starve-fed polymerization experiments

Table S2. Conversions, molecular weight and dispersity trends of the multi-armed poly(lactide)s monitored over a 24h polymerization period through the described monomer-starved method. Monomer concentration was kept constant at 1M and a [DBU]:[OH] molar ratio of 1 was used. All polymerization were then precipitated after 6-hours, to avoid undesired increase in dispersity due to the enhanced occurrence of termination reactions at higher polymerization periods.

Monomer	Initiator	Reaction extent (h)	# arms	[M]:[I] (DP_{tot})	Conversion (%)[a]	Mn_{NMR} (kDa)[c]	Mn_{GPC} (kDa)[c]	Mw_{GPC} (kDa)[c]	Đ[c]
L-LA	BnOH	1.5	1	50	49 (98)	7.1	7.1	7.4	1.04
		3		50	50 (99)	7.2	7.6	8.8	1.16
L-LA		4.5		100	87	12.5	12.9	13.9	1.08
		6		100	88	12.7	12.7	14.4	1.13
		24		100	91	13.1	11.8	14.8	1.25
L-LA	BDM	1.5	2	100	47 (93)	13.5	14.5	15.4	1.06
		3		100	49 (97)	14.1	14.3	15.0	1.05
L-LA		4.5		200	81	23.3	23.2	24.1	1.04
		6		200	88	25.3	24.0	25.5	1.06
		24		200	93	26.8	26.7	27.7	1.04
L-LA	PET	1.5	4	200	38 (76)	21.9	24.0	24.3	1.01
		3		200	48 (96)	27.6	27.1	28.6	1.06
L-LA		4.5		400	82	47.2	44.4	45.3	1.02
		6		400	86	49.5	45.2	49.7	1.10
		24		400	80	46.1	43.6	50.7	1.16
L-LA	diPET	1.5	6	300	39 (78)	33.7	32.3	33.6	1.04
		3		300	48 (95)	41.5	39.3	45.9	1.17
L-LA		4.5		600	79	68.3	59.2	73.7	1.24
		6		600	89	76.9	73.6	76.5	1.04
		24		600	88	76.0	74.0	78.6	1.06
L-LA	Sucr	1.5	8	400	38 (76)	43.2	44.3	44.8	1.01
		3		400	41 (82)	47.3	47.2	51.5	1.09
L-LA		4.5		800	75	86.4	80.2	82.8	1.03
		6		800	80	92.4	86.8	88.9	1.02
		24		800	81	93.3	85.0	90.7	1.07
L-LA	BnOH	1.5	1	50	41 (82)	5.9	5.9	6.2	1.05
		3		50	49 (98)	7.1	7.3	7.9	1.08
DL-LA		4.5		100	65	9.3	10.1	10.8	1.07
		6		100	81	11.5	11.9	12.3	1.03
		24		100	75	10.8	10.8	11.3	1.05
L-LA	BDM	1.5	2	100	46 (87)	13.1	12.7	13.5	1.06
		3		100	49 (98)	14.2	14.3	15.6	1.09
DL-LA		4.5		200	65.2	18.8	19.5	21.5	1.10
		6		200	81.6	23.5	22.9	24	1.05

		24		200	79.1	22.8	22.3	22.9	1.03
L-LA	PET	1.5	4	200	42 (85)	24.4	23.2	24.9	1.07
		3		200	49 (97)	28.1	27.2	29.3	1.08
DL-LA		4.5		400	67	38.7	41.3	45.1	1.09
		6		400	79	45.4	44.9	47.5	1.06
		24		400	77	44.5	43.7	47.8	1.09
L-LA	diPET	1.5	6	300	41 (82)	35.3	35.2	37.9	1.08
		3		300	49 (97)	42.0	42.3	52.4	1.24
DL-LA		4.5		600	62	54.0	56.6	59.4	1.05
		6		600	76	65.7	69.4	74.0	1.07
		24		600	76	65.8	64.8	77.1	1.19
L-LA	Sucr	1.5	8	400	38 (76)	44.2	46.6	47.2	1.01
		3		400	41 (82)	47.6	45.7	47.0	1.03
DL-LA		4.5		800	71	81.8	78.2	81.2	1.04
		6		800	77	89.0	82.3	82.8	1.01
		24		800	75	86.4	80.6	85.6	1.06
DL-LA	BnOH	1.5	1	50	40 (80)	5.8	6.8	7.9	1.16
		3		50	48 (96)	6.9	7.5	7.9	1.05
L-LA		4.5		100	70	10.1	9.1	9.8	1.08
		6		100	79	11.4	10.0	11.4	1.14
		24		100	77	11.1	11.6	12.5	1.08
DL-LA	BDM	1.5	2	100	42 (83)	12.0	12.4	13.1	1.06
		3		100	47 (95)	13.7	14.0	15.6	1.11
L-LA		4.5		200	68	19.6	19.7	20.9	1.06
		6		200	80	22.9	21.5	24.7	1.15
		24		200	78	22.6	21.2	23.3	1.10
DL-LA	PET	1.5	4	200	40 (80)	23.0	21.6	23.5	1.09
		3		200	48 (95)	27.3	27.2	27.8	1.02
L-LA		4.5		400	68	38.9	38.0	39.5	1.04
		6		400	78	45.1	42.1	46.5	1.10
		24		400	77	44.5	44.2	45.4	1.03
DL-LA	diPET	1.5	6	300	36 (72)	31.2	31.2	31.6	1.01
		3		300	46 (92)	39.7	39.1	47.4	1.21
L-LA		4.5		600	68	59.1	57.4	60.2	1.05
		6		600	77	66.9	69.7	77.3	1.11
		24		600	75	64.8	63.5	74.8	1.18
DL-LA	Sucr	1.5	8	400	32 (64)	37.0	38.5	38.8	1.01
		3		400	40 (80)	46.3	42.3	46.5	1.10
L-LA		4.5		800	73	84.1	76.8	80.9	1.05
		6		800	79	91.2	79.3	89.0	1.12
		24		800	78	90.0	77.2	88.5	1.15
DL-LA	BnOH	1.5	1	50	43 (85)	6.2	7.8	8.2	1.05
		3		50	48 (96)	6.9	7.5	8.5	1.13
DL-LA		4.5		100	75	10.8	10.0	11.9	1.19
		6		100	84	12.1	11.8	13.4	1.14

		24		100	80	11.5	11.9	14.8	1.24
DL-LA	BDM	1.5	2	100	43 (86)	12.4	14.5	15.0	1.03
		3		100	49 (97)	14.1	14.3	15.0	1.05
DL-LA		4.5		200	75	21.6	17.6	18.1	1.03
		6		200	83	23.9	21.3	23.4	1.10
		24		200	79	22.8	24.6	26.1	1.06
DL-LA	PET	1.5	4	200	34 (68)	19.6	19.8	20.2	1.02
		3		200	48 (96)	27.6	25.7	27.0	1.05
DL-LA		4.5		400	71	40.9	35.1	38.3	1.09
		6		400	82	47.2	37.3	42.5	1.14
		24		400	76	43.8	41.1	43	1.05
DL-LA	diPET	1.5	6	300	37 (74)	32.0	32.8	37.1	1.13
		3		300	45 (90)	38.9	36.6	45.3	1.24
DL-LA		4.5		600	71	61.3	55.1	59.6	1.08
		6		600	79	68.3	64.4	70.6	1.10
		24		600	74	63.9	65.9	70.0	1.06
DL-LA	Sucr	1.5	8	400	36 (72)	41.4	40.8	41.7	1.02
		3		400	39 (79)	45.3	41.1	44.4	1.08
DL-LA		4.5		800	70	80.6	73.6	75.8	1.03
		6		800	76	87.4	76.5	81.1	1.06
		24		800	68	78.0	75.3	80.2	1.07

Sampling for analysis was performed always prior new monomer addition. [a]Percentage relative to monomer measured by ^{1}H NMR in $CHCl_3$. Conversion values with respect to current amount of monomer (DP_{arm} of 50) reported in parenthesis. [b]Measured by ^{1}H NMR in $CHCl_3$ by calculating the corresponding monomer conversion for each time point. [c]MW data for the obtained monomodal distributions measured by GPC in THF using triple detection.

Table S3: dn/dc values for all PLAs as measured by triple-detection GPC/SEC in THF.

Sample	Arms	dn/dc (mL/g)
*L*_(PLLA)	1	0.040
*L*_$(PLLA)_2$	2	0.041
*S*_$(PLLA)_4$	4	0.042
*S*_$(PLLA)_6$	6	0.042
*S*_$(PLLA)_8$	8	0.043
*C*_$(PLLA)_{140}$	~140	0.045
*L*_(PLLA-*b*-PDLLA)	1	0.039
*L*_(PLLA-*b*-PDLLA)$_2$	2	0.040
*S*_(PLLA-*b*-PDLLA)$_4$	4	0.043
*S*_(PLLA-*b*-PDLLA)$_6$	6	0.042
*S*_(PLLA-*b*-PDLLA)$_8$	8	0.043
*C*_(PLLA-*b*-PDLLA)$_{140}$	~140	0.044
*L*_(PDLLA-*b*-PLLA)	1	0.039
*L*_(PDLLA-*b*-PLLA)$_2$	2	0.041
*S*_(PDLLA-*b*-PLLA)$_4$	4	0.042
*S*_(PDLLA-*b*-PLLA)$_6$	6	0.043
*S*_(PDLLA-*b*-PLLA)$_8$	8	0.043
*C*_(PDLLA-*b*-PLLA)$_{140}$	~140	0.045
*L*_(PDLLA)	1	0.040
*L*_$(PDLLA)_2$	2	0.040
*S*_$(PDLLA)_4$	4	0.042
*S*_$(PDLLA)_6$	6	0.042
*S*_$(PDLLA)_8$	8	0.043
*C*_$(PDLLA)_{140}$	~140	0.049

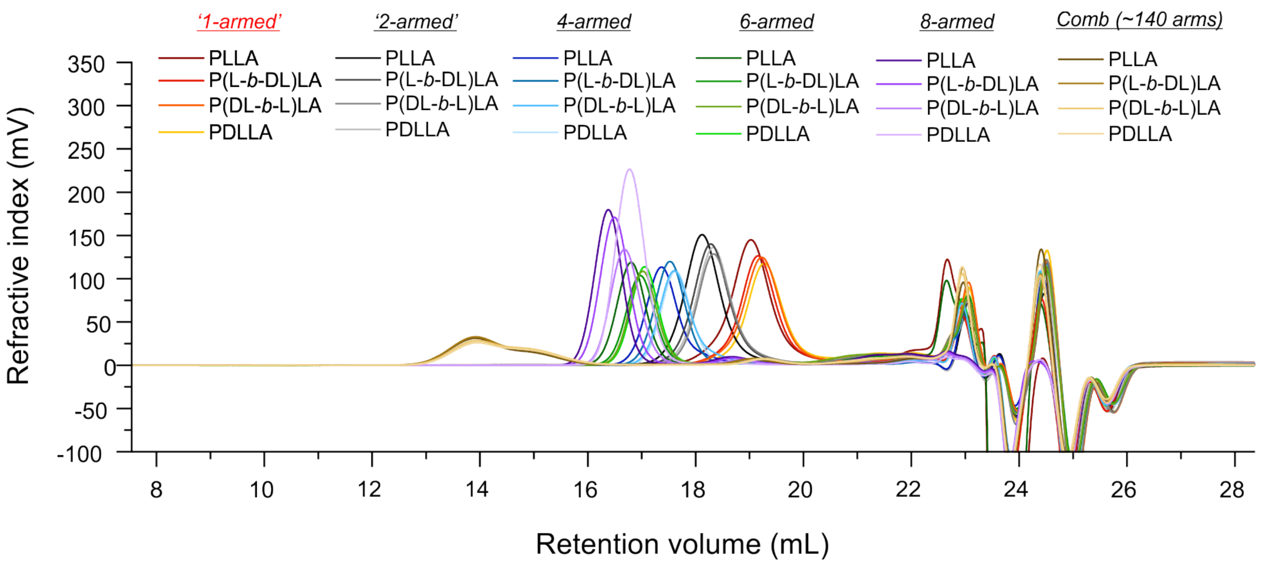

Figure S3. Complete GPC traces (RI detector) for all PLAs produced in this study. Please note that a) the solvent front is at >22 mL, b) the exclusion limit is at about 14 mL (see also Figure S4); the GPC traces showing a wide peak extending up to that limit are those of the comb polymers (see again Figure S4).

S4. Analysis of tacticity

Table S4. Probability of finding a racemic P*r* (ranging between 0 for fully isotactic and 0.5 for atactic PLA) was and percentage of racemization (%rac; contribution of *mmmrr* sequence signal to the total polymer stereoconfiguration).

Sample	Pr [a]	% *rac (% mmmrr)* [b]
*L*_(PLLA)	0.13	6.3
*L*_$(PLLA)_2$	0.13	4.8
*S*_$(PLLA)_4$	0.12	4.4
*S*_$(PLLA)_6$	0.14	3.6
*S*_$(PLLA)_8$	0.18	3.4
*C*_$(PLLA)_{140}$	0.22	2.2
*L*_(PLLA-*b*-PDLLA)	0.16	4.7
*L*_$(PLLA\text{-}b\text{-}PDLLA)_2$	0.23	2.9
*S*_$(PLLA\text{-}b\text{-}PDLLA)_4$	0.23	2.5
*S*_$(PLLA\text{-}b\text{-}PDLLA)_6$	0.22	3.0
*S*_$(PLLA\text{-}b\text{-}PDLLA)_8$	0.27	1.8
*C*_$(PLLA\text{-}b\text{-}PDLLA)_{140}$	0.28	2.6
*L*_(PDLLA-*b*-PLLA)	0.30	2.3
*L*_$(PDLLA\text{-}b\text{-}PLLA)_2$	0.33	1.8
*S*_$(PDLLA\text{-}b\text{-}PLLA)_4$	0.29	1.4
*S*_$(PDLLA\text{-}b\text{-}PLLA)_6$	0.32	1.6
*S*_$(PDLLA\text{-}b\text{-}PLLA)_8$	0.32	0.9
*C*_$(PDLLA\text{-}b\text{-}PLLA)_{140}$	0.33	0.7
*L*_(PDLLA)	0.37	2.1
*L*_$(PDLLA)_2$	0.36	1.3
*S*_$(PDLLA)_4$	0.36	1.5
*S*_$(PDLLA)_6$	0.35	0.7
*S*_$(PDLLA)_8$	0.36	0.8
*C*_$(PDLLA)_{140}$	0.37	0.9

[a]Calculated following the equation $\mathrm{Pr} = \sqrt{(2 \cdot [rmr])}$, where $[rmr]$= Area (*rmr*)/Area (tot). [b] %rac corresponds to Area (*mmmrr*)/Area(tot)·100

S5. Characterization of the PVA-initiated comb poly(lactide)s

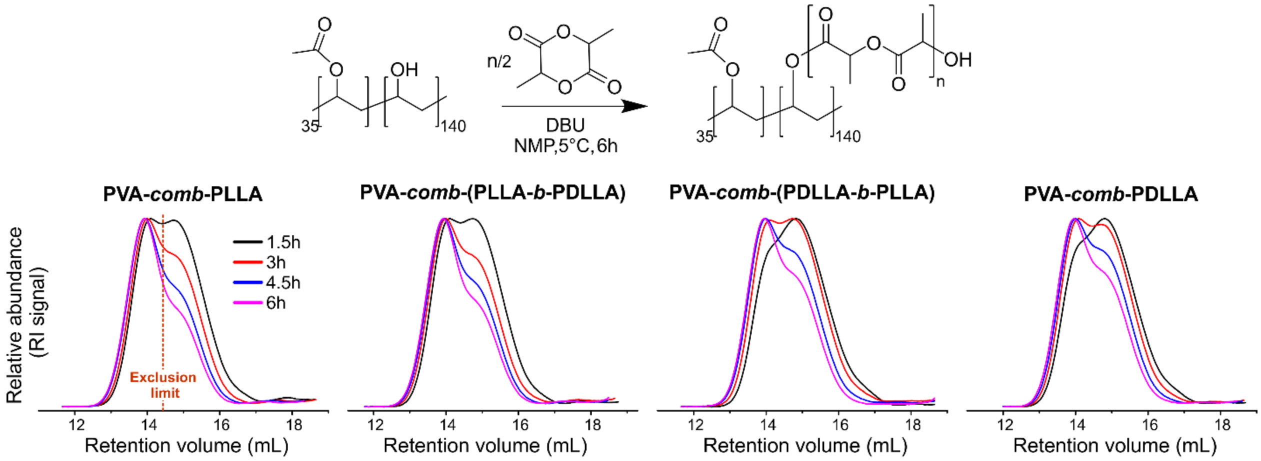

Sample	Mn_{GPC} (kDa)[a]	Mw_{GPC} (kDa)[a]	Đ[a]
*C*_(PLLA)$_{140}$	1467.0	2206.2	1.5
*C*_(PLLA-*b*-PDLLA)$_{140}$	1330.0	2007.7	1.5
*C*_(PDLLA-*b*-PLLA)$_{140}$	1372.8	2026.2	1.5
*C*_(PDLLA)$_{140}$	1316.1	2072.5	1.6

Poly(vinyl alcohol) 80% hydrolized (Mn=15KDa, Đ = 1,3).[a]Average MW data for the obtained multimodal distributions measured by GPC in THF using

Figure S4. Scheme of the PVA-initiated LA polymerization (*above*), GPC chromatograms (normalized refractive index (RI) signal, *middle*) and MW data of the polymers obtained after a 6-hour, low-temperature and starve-fed polymerization. Please note that the MW data are reported for completeness, but - due to significant portions of the MW distribution falling above the total exclusion limit of the columns – they do not provide a reliable assessment of the distribution itself

Table S5: Results of multi-angle static light scattering (MALS) analysis for PVA-*comb*-PLA structures.

Sample	$\overline{M_w}$ (kDa)	Rg (nm)	A2 (mol·mL/g^2)
*C*_(PLLA)$_{140}$	1913 ± 38	21 ± 3	(1.49 ± 0.12)·10^{-5}
*C*_(PLLA-*b*-PDLLA)$_{140}$	2033 ± 69	18 ± 5	(1.45 ± 0.20)·10-^{5}
*C*_(PDLLA-*b*-PLLA)$_{140}$	1673 ± 82	21 ± 6	(1.80 ± 0.36)·10^{-5}
*C*_(PDLLA)$_{140}$	1229 ± 34	22 ± 4	(2.74 ± 0.28)·10^{-5}

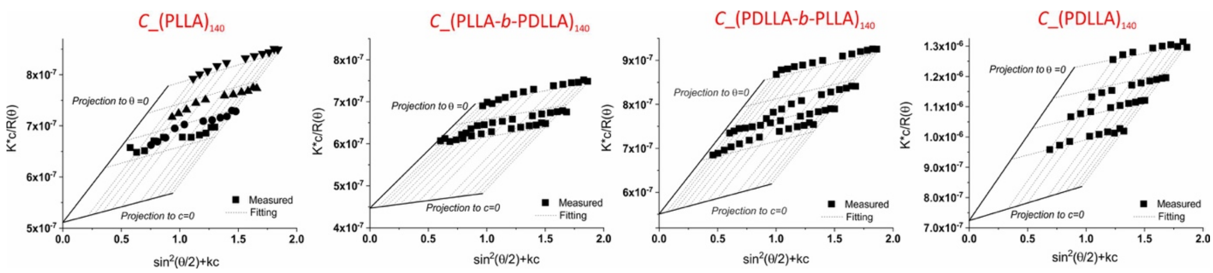

Figure S5. Representative Zimm plots for the comb polymers

S6. Thermal analysis

Table S6. Thermal properties of the multi-armed poly(lactide)s measured by DSC.

Structure	# arms	Tg (°C)[a]	Tm_{onset} (°C)[b]	Tm_{peak} (°C)[b]	Xc (%)[c]
PLLA	1	50	77	132	35
	2	52	98	133	32
	4	53	84	130	27
	6	53	82	125	12
	8	53	104	118	6
	140	55	-	-	-
PLLA-*b*-PDLLA	1	48	-	-	-
	2	50	106	-	7
	4	52	-	-	-
	6	52	-	-	-
	8	53	-	-	-
	140	53	-	-	-
PDLLA-*b*-PLLA	1	47	-	-	-
	2	49	-	-	-
	4	51	-	-	-
	6	51	-	-	-
	8	50	-	-	-
	140	52	-	-	-
PDLLA	1	47	-	-	-
	2	50	-	-	-
	4	51	-	-	-
	6	52	-	-	-
	8	52	-	-	-
	140	52	-	-	-

[a] Glass transition temperature (Tg) was calculated by half-height method from DSC 2nd heating scan.
[b] Melting temperatures as measured from DSC 1st heating scan.
[c] Calculated as $X_C = (|\Delta H_{cc} - \Delta H_m|/\Delta H_m^0) \cdot 100$, where ΔH_m^0= 93 J/g (100% crystalline PLLA)[21] and $\Delta H_{cc} \neq 0$ only for 2-armed PLLA, where a value the DSC software (TRIOS) recognized a cold crystallization occurring between 90 and 115°C, with ΔH_{cc}=2 J/g. For comparison the melting enthalpy is ΔH_m= 32 J/g, in the area 115-140°C.

S7. Shear rheometry

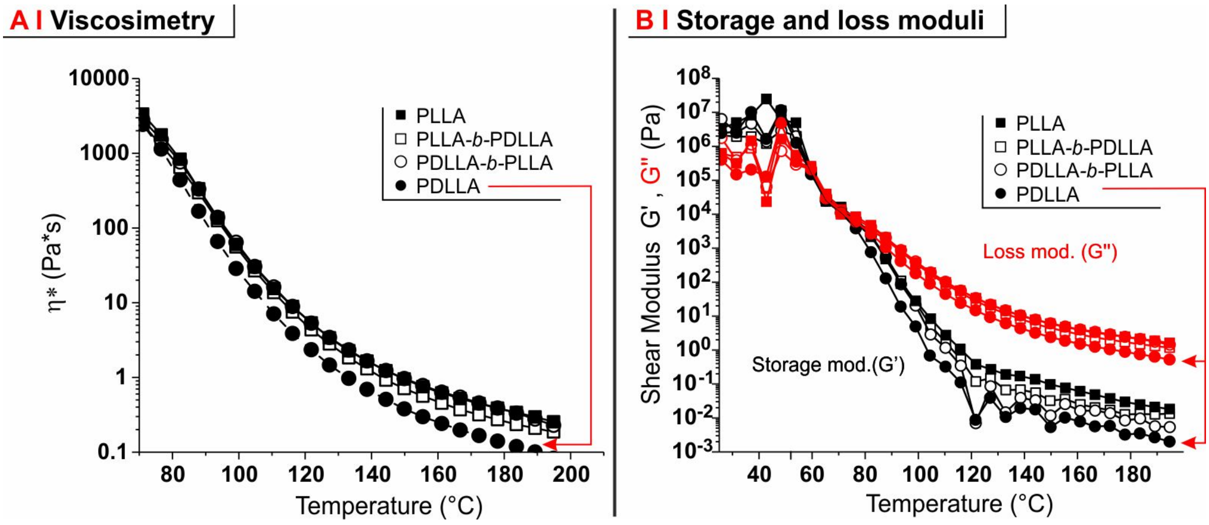

Figure S6. ***A.*** Complex shear viscosity (η^*) dependency on temperature for 6-armed PLAs with different tacticity, in stress-controlled oscillatory experiments (parallel-plate geometry, oscillation frequency: 1Hz). Temperature was decreased from 200 to 25 °C at 5 °C/min. ***B.*** Storage (G', black symbols) and loss (G", red symbols) shear moduli for the same polymers. It is apparent that – upon cooling - both G' and G" stabilize below the Tg, while a region of G'~G" (a rubbery plateau) is present above; of note, a crystallization does not take place during the relatively rapid cooling, although a the temperature dependence of both moduli changes around what would be the Tm.

S8. Nanoindentation via AFM

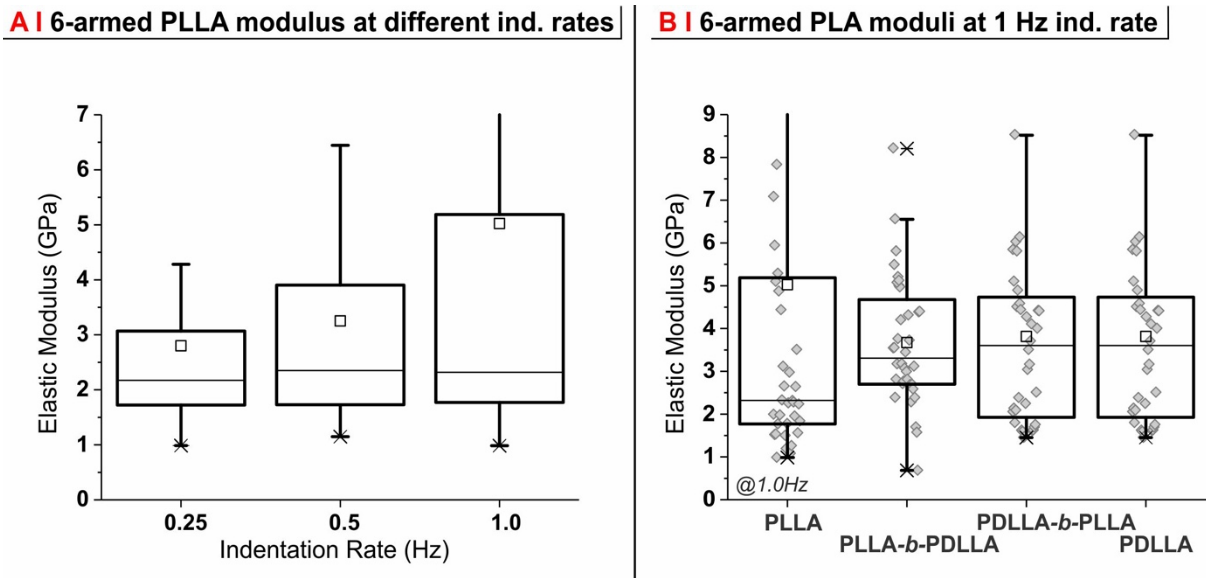

Figure S7. ***A.*** The speed of indentation rate (here increased from 0.25Hz to 1.0Hz) increased the value recorded for the elastic modulus. ***B.*** Using an indentation rate of 1.0 Hz, no difference or trend was observed in the elastic modulus of the different samples.

S8. References

1. Armstrong, J. K.; Wenby, R. B.; Meiselman, H. J.; Fisher, T. C. The hydrodynamic radii of macromolecules and their effect on red blood cell aggregation. *Biophysical Journal* **2004,** 87 (6), 4259-4270 DOI: 10.1529/biophysj.104.047746.
